# Complex History and Differentiation Patterns of the *t*-Haplotype, a Mouse Meiotic Driver

**DOI:** 10.1534/genetics.117.300513

**Published:** 2017-11-14

**Authors:** Reka K. Kelemen, Beatriz Vicoso

**Affiliations:** Institute of Science and Technology Austria, 3400 Klosterneuburg, Austria

**Keywords:** meiotic driver, t-haplotype, genome evolution

## Abstract

The *t*-haplotype, a mouse meiotic driver found on chromosome 17, has been a model for autosomal segregation distortion for close to a century, but several questions remain regarding its biology and evolutionary history. A recently published set of population genomics resources for wild mice includes several individuals heterozygous for the *t*-haplotype, which we use to characterize this selfish element at the genomic and transcriptomic level. Our results show that large sections of the *t*-haplotype have been replaced by standard homologous sequences, possibly due to occasional events of recombination, and that this complicates the inference of its history. As expected for a long genomic segment of very low recombination, the *t*-haplotype carries an excess of fixed nonsynonymous mutations compared to the standard chromosome. This excess is stronger for regions that have not undergone recent recombination, suggesting that occasional gene flow between the *t* and the standard chromosome may provide a mechanism to regenerate coding sequences that have accumulated deleterious mutations. Finally, we find that *t*-complex genes with altered expression largely overlap with deleted or amplified regions, and that carrying a *t*-haplotype alters the testis expression of genes outside of the *t*-complex, providing new leads into the pathways involved in the biology of this segregation distorter.

MEIOTIC drivers (also known as segregation distorters) are selfish alleles or chromosome variants that can transmit themselves to over 50% of the progeny of heterozygous individuals (Burt and Trivers 2009; Lindholm *et al.* 2016), often by killing or inactivating gametes that carry the nondriver allele. This requires the combined action of at least one distorter gene, which attacks gametes, and a responder gene, which protects gametes carrying the driver [reviewed in Lindholm *et al.* (2016)]. Linkage between the distorter and responder genes is required for the survival of the driver, and successful drivers often arise in regions of low recombination (Schwander *et al.* 2014). Conversely, the presence of drivers can select for reduced recombination around the driving and responding loci (Charlesworth and Hartl 1978). Autosomal drivers usually have no detectable phenotypic effects, and much of what is known about them comes primarily from studies of two model systems: Segregation Distorter in *Drosophila melanogaster* (Larracuente and Presgraves 2012) and the *t*-haplotype of the domestic mouse *Mus musculus*.

The *t*-haplotype is a 40-Mb variant of the proximal portion of chromosome 17 (Burt and Trivers 2009; Herrmann and Bauer 2012), which shows suppressed recombination with the standard chromosome due to the accumulation of several inversions (three on the *t*-haplotype and one on the standard chromosome; Artzt *et al.* 1982; Herrmann *et al.* 1986; Burt and Trivers 2009).When present in females, it is transmitted to 50% of the progeny, but > 90% of the progeny of *t*-carrying males inherit it (Chesley and Dunn 1936; Herrmann and Bauer 2012). Despite this strong driving capacity, *t*-haplotypes remain at relatively low frequency (10–25%; Ardlie 1998), partly because individuals carrying two copies of the *t*-haplotype have strongly reduced fertility and viability (Herrmann and Bauer 2012). *t*-haplotypes are found throughout the *M. musculus* species complex (which includes *M. m. domesticus*, *M. m. musculus*, and *M. m. castaneus*), but not in the close outgroup *M. spretus* (Lyon 2003).

The genetics of transmission distortion of the *t*-haplotype are well understood, and several drivers, as well as one responder, have been identified. These lead to morphological defects in spermatozoa that do not carry a *t*-haplotype due to excessive activation of the chromosome 17 gene *Smok* (Bauer *et al.* 2007, 2012; Herrmann and Bauer 2012). By contrast, with one exception (Sugimoto 2014), the loci responsible for the lethality and sterility of homozygous *t*-haplotypes have not been mapped to specific genes. It is further unclear if these are caused by single loci in each *t*-haplotype, or by the accumulation of many deleterious mutations [but see Howell *et al.* (2004) for at least one example of a cryptic lethal mutation]. Selection is ineffective in regions of low recombination and genes located in such regions often accumulate deleterious mutations (Woolfit 2009; Campos *et al.* 2014). Nonrecombining segregation distorters should be particularly affected (Dyer *et al.* 2007), as mutations that arise there can spread if their harmful effect does not outweigh the selective advantage of the linked driver, and new mutations that increase driving efficiency can sweep linked deleterious variants to fixation (Presgraves *et al.* 2009). The extent to which the hundreds of genes on the *t*-haplotype have deteriorated, and whether occasional recombination with the standard chromosome is sufficient to maintain genetic integrity in meiotic drivers over millions of years (Dyer *et al.* 2007; Pieper and Dyer 2016), remain open questions.

Several questions also remain regarding the origin and sequence evolution of this meiotic driver. Sequence divergence between the *t*-haplotype and the standard chromosome 17 led to the conclusion that the first inversion arose over 3 MYA, and inversion 4 ∼1.5 MYA (Hammer and Silver 1993). While these are likely overestimates given the current *M. spretus/M. musculus* estimates of divergence (Harr *et al.* 2016), they clearly precede the origin of all the *M. musculus* subspecies in which they are found (White *et al.* 2009), showing that they were present in the ancestral population. However, the *t*-haplotype sequences of the different subspecies show very little differentiation between them, suggesting that a single *t*-haplotype introgressed < 0.1 MYA throughout the species group (Morita *et al.* 1992; Hammer and Silver 1993). Much of this early work relied on short sequences and it is unclear if these patterns capture the full history of this driver; further, where this haplotype introgressed from is still unknown. Inversions 3 and 4 have been found to carry more genetic variants than inversion 2, with occasional recombination between different *t*-haplotypes (Dod *et al.* 2003), but also with standard chromosomes (Herrmann *et al.* 1987; Erhart *et al.* 1989, 2002; Hammer *et al.* 1991; Wallace and Erhart 2008) likely playing a role in their differentiation. How this varies throughout each inversion is unclear, something that is potentially problematic, as regions closer to breakpoints generally show a stronger reduction in recombination than the middle of inversions (Wallace and Erhart 2008). It has therefore not been excluded that differences between inversions could represent a sampling bias rather than a real difference in their age, or that estimates of the age of inversions have been biased by secondary recombination events.

Here, we take advantage of a recently published population genomics data set of wild mice (Harr *et al.* 2016), which contains RNA-sequencing (RNA-seq) and genomic data derived from 15 *M. musculus t*-haplotype carriers, 32 noncarriers from the same populations and eight individuals of the closely related species *M. spretus*, to characterize the *t*-haplotype at both the genomic and gene expression level.

## Materials and Methods

### Data source

Harr *et al.* (2016) recently published extensive population genomics resources for three subspecies of *M. musculus*, as well as its close outgroup *M. spretus*. These included 15 individuals heterozygous for *t*-haplotypes [four in *M. m. domesticus*, eight in *M. m. musculus* (excluding mouse CR29 that we suspect to be a partial *t*-haplotype-carrier), and three in *M. m. castaneus*], as well as many noncarriers [see Table 1 of Harr *et al.* (2016)]. For each individual, we downloaded a BAM alignment file with reads mapped to the house mouse reference genome and the respective variant-containing variant call format (VCF) file from http://wwwuser.gwdg.de/∼evolbio/evolgen/wildmouse/.

Harr *et al.* (2016) further generated RNA-seq reads for brain, liver, spleen, heart, thyroid, kidney, and testis of the same 16 *M. m. domesticus* specimens that were used for genomic sequencing. The RNA-seq reads were downloaded from the National Center for Biotechnology Information Short Reads Archive (bioproject PRJEB11897).

A detailed protocol of all the steps involved and code used in our analysis is provided in Supplemental Material, File S1, while supplementary figures, tables, and data are provided in File S2.

### Copy number variant (CNV) detection

To avoid biases caused by polymorphic or *t*-specific CNVs, we called CNVs using the software Control-FREEC (Boeva *et al.* 2012) and combined these with the list of CNVs that Harr *et al.* (2016) obtained using the software CNVnator; both methods rely on differences in genomic coverage to detect deletions or duplications. We first ran Control-FREEC on each of the 55 BAM files against the reference genome, using window sizes of 1 and 5 kb. To fully detect *t*-specific CNVs, we then used Control-FREEC to call CNVs between the pooled *t*-carrier mice of each subspecies and four randomly chosen non-*t*-carrier mice controls from the same subspecies (see details in the supplemental methods described in File S1). A genomic region was classified as a CNV if it was detected in at least one sample by either of the two software packages.

### SNP filtering

We downloaded the two multisample VCF files provided by Harr *et al.* (2016). One contained the high-quality variants obtained by GATK’s VSQR filtering, while the other contained the unfiltered raw SNPs. We conducted our entire analysis on both variant sets, and used BCFtools (Li 2011) to handle the VCF files.

**Figure 1:**
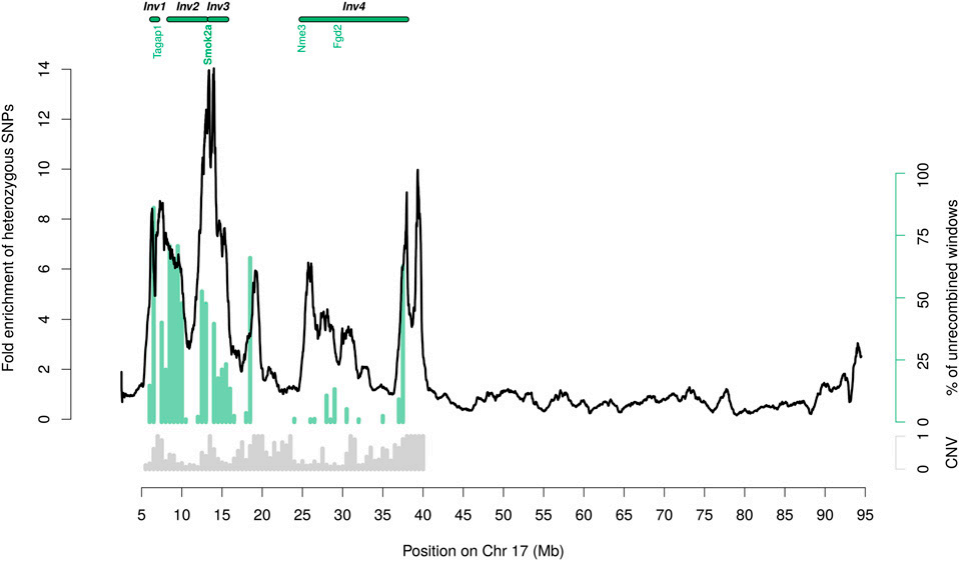
Heterozygosity levels and phylogenetic topology along the *t*-haplotype of *M. m. domesticus*. The black line shows the heterozygous SNP density of *t*-carrier mice divided by the heterozygous SNP density of control noncarrier mice. The ratio is averaged over 1-Mb windows (sliding by 1-kb). Gray bars below show, for each 0.5-Mb segment of the *t*-complex, the proportion that was identified as a CNV in any of the 55 mice. Green bars indicate in each 0.5-Mb segment the proportion of trees that show all *M. m. domesticus t*-haplotypes outside of the *M. musculus* species cluster (see Figure 2 and *Materials and Methods*). We plotted the data for the entire chromosome 17 without masking CNVs. Chr, Chromosome; CNV, copy number variant; Inv, inversion.

**Figure 2:**
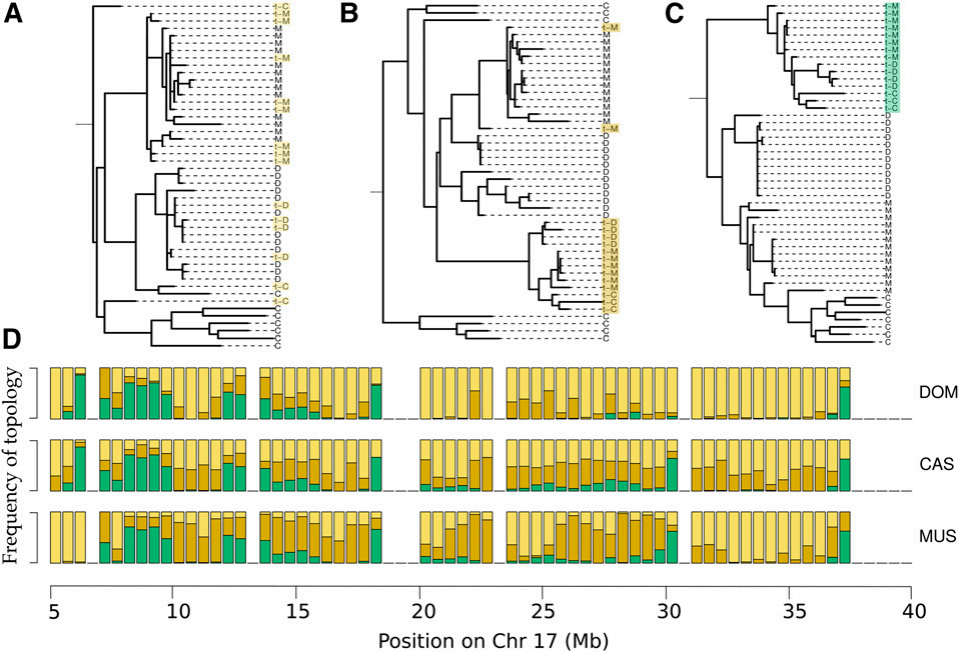
Extent of recombination along the *t*-haplotype. (A–C) Example trees showing topologies that suggest very recent (A), recent (B), or no (C) recombination between the *t*-haplotype and the standard chromosome. In each tree, D, C, M, and S, respectively, represent noncarriers of *M. m domesticus*, *M. m. castaneus*, *M. m. musculus*, and *M. spretus*, while t-D, t-C, and t-M represent pseudo-*t*-haplotypes of *M. m domesticus*, *M. m. castaneus*, and *M. m. musculus*. Topology A is denoted with yellow, B with orange, and C with green. (D) The proportion of trees (obtained from nonoverlapping 5-kb windows for region 5–40 Mb of chromosome 17) with topologies A, B, and C for the *t*-haplotypes of each of the subspecies (DOM for *M. m. domesticus*, CAS for *M. m. castaneus*, and MUS for *M. m. musculus*). The proportion is shown for each 500-kb nonoverlapping region along the *t*-complex (5–40 Mb on chromosome 17).

**Figure 3:**
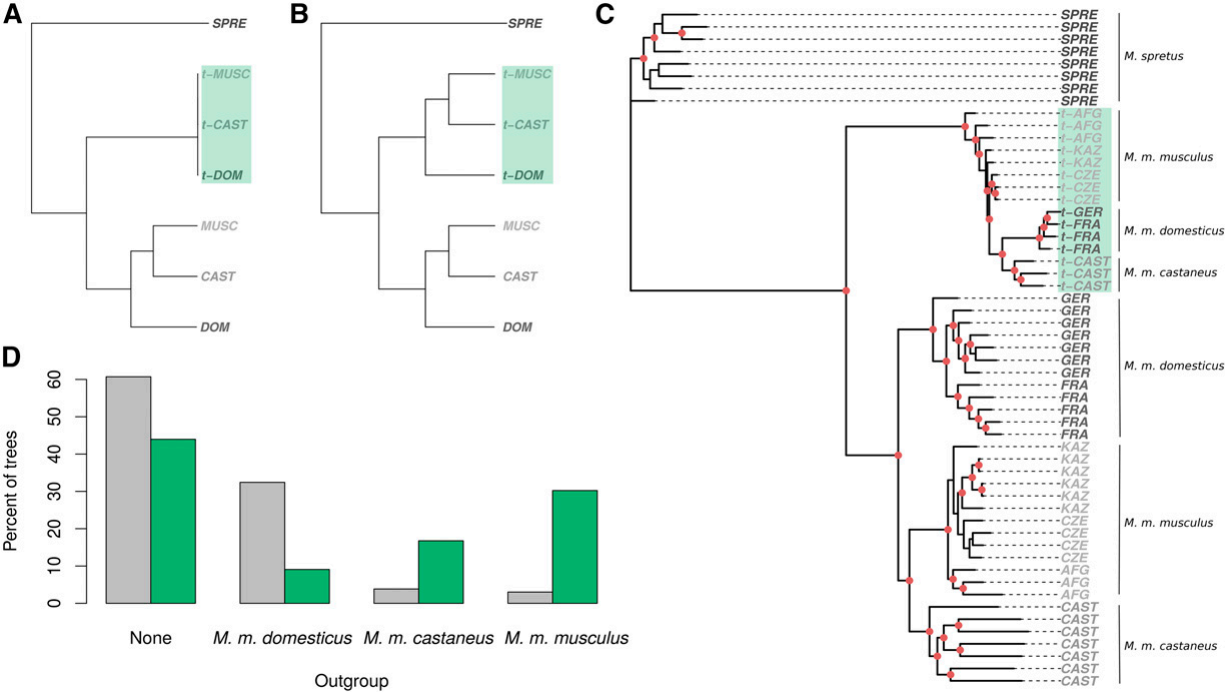
Recent history of the *t*-haplotype. (A) Model phylogeny under the scenario of recent introgression of a single *t*-haplotype into all *M. musculus* subspecies. (B) Model phylogeny under the hypothesis of independent maintenance of ancestrally present *t*-haplotypes in the different subspecies. CAST, DOM, MUSC, and SPRET represent noncarriers of *M. m. castaneus*, *M. m. domesticus*, *M. m. musculus*, and *M. spretus*, respectively, while t-CAST, t-DOM, and t-MUSC represent *t*-haplotypes of *M. m. castaneus*, *M. m domesticus*, and *M. m. musculus*, respectively. (C) Phylogeny of pseudo-*t*-haplotypes and noncarrier mice from the three *M. musculus* subspecies and the sister species, *M. spretus*. Nodes with bootstrap values > 94% are marked with red dots. Only regions of the *t*-complex where no recombination between the standard chromosomes and the *t*-haplotype could be detected were included (green regions in Figure 2). Sequences starting with “t-” (highlighted with a green background) refer to *t*-haplotypes. AFG, CZE, and KAZ stand for *M. m. musculus* from Afghanistan, the Czech Republic, and Kazakhstan; GER and FRA for *M. m. domesticus* from Germany and France; CAST stands for *M. m. castaneus*; and SPRE for *M. spretus*. (D) Percentage of 5-kb windows without recombination for which the resulting phylogeny yields one subspecies as the outgroup to the others (“none” shows the proportion of windows for which no subspecies was an outgroup). Gray bars represent the phylogeny of non-*t*-carriers, and green bars represent the phylogeny of pseudo-*t*-haplotypes (see *Materials and Methods*).

**Figure 4:**
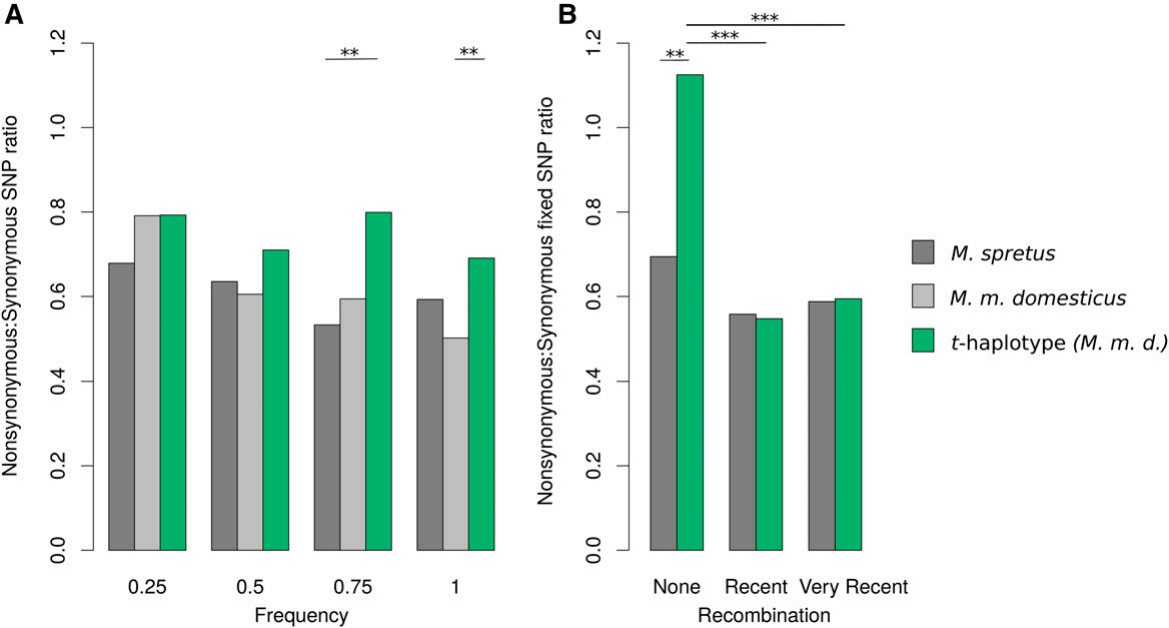
Nonsynonymous to synonymous (NS/S) SNP ratio in the *t*-complex region. (A) NS/S for SNPs found at different frequencies in the pseudo-*t*-haplotypes (green), in the non-*t*-carrier control *M. m. domesticus* (light gray), and in *M. spretus* individuals (dark gray) for the *t*-complex. (B) NS/S for *t*-haplotype (green) and *M. spretus* (dark gray) SNPs found in regions for which no recent recombination could be detected in *M. m. domesticus t*-haplotypes, recent recombination could be detected, and very recent and/or extensive recombination could be detected. The categories were determined based on the phylogenetic topologies shown in Figure 2.

We carried out our analysis using three different SNP-filtering procedures (detailed in File S1). In filtering procedure 1, we used the variants classified as PASS by Harr *et al.* (2016), and removed sites that were not SNPs, such as indels (Figure 1 and Figure S3 and Figure S4 in File S2). We further deleted sites located within CNVs for the phylogenetic and deterioration analyses (Figure 2, Figure 3, Figure 4, and Figure S6, Figure S7, Figure S8, and Figure S9 in File S2).

The raw variants were similarly filtered for CNVs and non-SNP variants, as well as additional criteria:

Filtering procedure 2: a variant was kept only if its total coverage was at least half of the average coverage for the given sample (reported in Table 1 of Harr *et al.* 2016); this yielded Figure S1 in File S2.
Filtering procedure 3: we used the same coverage filtering as in procedure 2, and additionally required that each heterozygous allele be supported by at ≥ 30% of the reads (Figure S2 in File S2).

### Estimates of heterozygosity in *M. m. domesticus*

We extracted variants for each *M. m. domesticus* sample from the multisample VCF file and retained only heterozygous sites. We then computed the average heterozygous SNP density of *t*-carrier mice in 1000-bp regions averaged over sliding 1-Mb windows. We plotted this density curve divided by the average of the corresponding densities computed in all *M. m. domesticus* non-*t*-carrier mice.

### Extracting “pseudo-*t*-haplotype” VCF files from heterozygous *t*-carriers

Given that *t*-carrier mice are heterozygous for the *t*-haplotype, SNPs found in their VCF files could represent *t*-derived variants or SNPs from their standard chromosome. SNPs that were homozygous in each *t*-carrier mouse were kept for further analysis, as they were likely present on both chromosomes. At heterozygous sites, we discarded all SNPs that were found in at least one noncarrier individual of any of the *M. musculus* subspecies and retained all others as putative *t*-haplotype SNPs. One caveat of this subtraction step is that it excluded any polymorphism that was present on both a *t*-haplotype and a standard chromosome, if it happened to be heterozygous in the *t*-carrier; however, given the low recombination rates between the *t* and the standard chromosome, there should be few shared segregating variants between the standard and *t*-haplotypes, such that these should be a minority. Conversely, rare genetic variants on the standard chromosome of *t*-carriers may be wrongfully retained as *t*-specific if they are not present in any of the noncarriers (but given the high level of *t*-to-standard chromosome divergence relative to genetic diversity in noncarriers, as shown in Figure 1, these should once again represent only a small minority of SNPs).

### Phylogenetic analysis

We examined the phylogeny of the 15 *t*-haplotypes from the three different *M. musculus* subspecies, along with the noncarrier mice from each population. SNP profiles from eight individuals from a closely related species, *M. spretus*, served as the outgroup.

We used the pseudo-*t*-haplotype SNP profiles (see previous section) to represent the 15 *t*-haplotypes of the carrier mice. We converted all VCF files to FASTA files using the mouse reference background with the *consensus* function of BCFtools (Li 2011), and concatenated all sequences into a multisample FASTA file. We subsampled this FASTA file into the desired genomic regions using the *faidx* function of SAMtools (Li *et al.* 2009). To compute the maximum likelihood phylogenies of the 15 *t*-haplotypes, and the 40 noncarriers from *M. m. domesticus*, *M. m. musculus*, *M. m. castaneus*, and *M. spretus*, we used the phylogenetic software IQTree (Nguyen *et al.* 2015) with an underlying Hasegawa–Kishono–Yano (HKY) model of DNA substitution. We assessed branch support values using an ultrafast bootstrap approximation UFBoot (Minh *et al.* 2013), which we iterated 1000 times. For computation of the maximum parsimony phylogenies, we used the software MEGA (Kumar *et al.* 2016) with default parameters but keeping only one tree, while for the neighbor-joining method we used FastPhylo with default parameters (Khan *et al.* 2013).

### Nonsynonymous to synonymous ratio (NS/S) of SNPs at varying frequencies

SNPs were classified as synonymous or nonsynonymous using the SNPeff software (Cingolani *et al.* 2012). We calculated the frequencies of each synonymous and nonsynonymous SNP in the four *M. m. domesticus* pseudo-*t*-haplotypes, in the 24 non-*t*-carrier *M. m. domesticus* chromosomes, and in the 16 *M. spretus* chromosomes using the genotypes provided in the VCF files. We counted a SNP once when it was found in a heterozygous individual, and twice when it was in a homozygous individual. In the case of the 24 non-*t*-carrier *M. m. domesticus* chromosomes, the four frequency classes were 1–6, 7–12, 13–18, and 19–24, while for the 16 *M. spretus* chromosomes, the frequency classes corresponded to 1–4, 5–8, 9–12, and 13–16. In the case of the four pseudo-*t*-haplotypes, the respective frequency classes were 1, 2, 3, and 4.

### Gene expression analysis

We obtained estimates of gene expression for each *M. m. domesticus* RNA-seq sample with Kallisto (Bray *et al.* 2016), using the *M. musculus* GRCm38.p4 coding sequence as reference. The resulting Kallisto transcript quantification was used as input for Sleuth, a software for differential expression analysis, using the gene aggregation feature (Pimentel *et al.* 2016).

### Data availability

All data analyzed in this study were previously published (Harr *et al.* 2016). The authors affirm that all analyses performed are fully described within the *Materials and Methods* and *Results* of the manuscript.

## Results

### Variable levels of divergence along the *t*-haplotype

We examined the extent of differentiation between the *t*-haplotype and the standard chromosome 17 of *M. m. domesticus*, for which there were four mice carrying the *t*-haplotype. While all the main figures are based on the high-quality SNPs provided by Harr *et al.* (2016), two alternative SNP-filtering procedures based on (1) coverage, and (2) coverage and allele frequency (see *Materials and Methods*), were used to check that results held independent of the filtering procedure (Figure S1 and Figure S2 in File S2).

We plotted the averaged SNP density of the four *t*-carrier mice along chromosome 17, divided by the respective average for noncarriers (for overlapping sliding windows of 1 Mb, Figure 1). Only heterozygous SNPs were used; in *t*-carriers these SNPs correspond to differences between the *t*-haplotype and the standard chromosome, whereas in control individuals they correspond to general levels of heterozygosity. Noncarrier individuals were used to control for variable genetic diversity rates along the chromosome (Figure S3 in File S2).

Figure 1 shows that *t*-carriers have increased heterozygosity in the region from 5 to 40 Mb of chromosome 17, consistent with the expected location of the *t*-haplotype. The excess of heterozygosity varies, with several regions showing a difference > 10-fold. Many of these also overlap with CNVs identified through differences in coverage between individuals (shown in gray in Figure 1), but these CNVs represent a subset of the high-divergence regions, so divergence in duplicated regions does not account for the increase in heterozygosity. These results hold when only intergenic and synonymous SNPs are used (Figure S4B in File S2) and when we compare instead the pseudo-*t*-haplotype SNP density (see *Materials and Methods*) to the SNP density of *M. spretus* (to control for fast-diverging regions, Figure S4, C–F in File S2). We also get a consistent pattern of divergence along the *t*-haplotype when we reproduce Figure 1 using insertions and deletions (Figure S5 in File S2).

We indicated the putative location of the four nonoverlapping inversions (Braidotti and Barlow 1997; Harrison *et al.* 1998; Herrmann *et al.* 1999; Zwart *et al.* 2001; Bauer *et al.* 2007, 2012; Sugimoto 2014) (inversions 1–4 in Figure 1), as well as the position of the genes known to be involved in transmission distortion. As expected, the largest peak of divergence is at the distal end of the second inversion (based on the standard sequence orientation), near the *Smok2A* gene, which is in the vicinity of the previously identified responder gene *Tcr* (Herrmann *et al.* 1999; *Tcr* itself is not present on the standard chromosome). This region is assumed to have been ancestrally recruited to the *t*-haplotype (Hammer and Silver 1993). However, inversion 4, which was hypothesized to have been acquired much later (Hammer and Silver 1993), contains peaks of nearly equally high divergence at ∼37 Mb. More generally, levels of divergence differ less between the inversions than they do within them, with putative inversion boundaries often coinciding with major peaks of divergence (Figure 1).

### Phylogenetic patterns along the *t*-haplotype suggest widespread recombination with the standard chromosome

While other factors could create a mosaic pattern of differentiation, the colocalization of high divergence and inversion boundaries suggests that recombination with the standard chromosome in the middle of inversions (Wallace and Erhart 2008) may have eroded the genetic differentiation of the *t*-haplotype, as has been suggested by several smaller-scale studies (Herrmann *et al.* 1987; Erhart *et al.* 1989, 2002; Hammer *et al.* 1991; Wallace and Erhart 2008). Such recombination events can be detected through changes in the phylogenetic topology of the *t*- and standard chromosomes of the *M. musculus* subspecies: segments of the *t*-haplotype that have not recombined since the split of the three subspecies should appear as an outgroup to them, while segments that have undergone recent recombination should cluster within the species group.

After removing SNPs from regions that were classified as CNVs, we created “pseudo-*t*-haplotype” SNP profiles from each of the VCF files of the 15 *t*-carrier mice provided by Harr *et al.* (2016). Since these mice are heterozygous for the *t*-haplotype and also carry a standard chromosome 17, we discarded heterozygous SNPs that were also found in any of the noncarrier individuals. Homozygous SNPs were presumed to be both on the standard chromosome and *t*-haplotype, and therefore kept even if also present in noncarriers.

After obtaining *t*-specific SNPs for each of the 15 *t*-carriers, we created *t*-haplotype sequences by replacing these SNPs into the *M. m. domesticus* reference genome. We did the same for each of the noncarrier mice (12 *M. m. domesticus*, 13 *M. m. musculus*, 7 *M. m. castaneus*, and 8 *M. spretus* individuals), for which the list of variants was supplied by Harr *et al.* (2016). To verify the reliability of our pipeline, we applied it to a region of the gene *Tcp-1* for which *t*-haplotype and standard sequences have been published (Morita *et al.* 1992; Figure S6 in File S2). Of the 24 published *t*-specific SNPs, 22 (92%) were recovered on our pseudo-*t*-haplotypes.

Using the HKY nucleotide substitution model of the phylogenetic software IQTree, we estimated the phylogenetic topology of the 15 *t*-haplotypes and 40 noncarriers in nonoverlapping 5-kb windows along the *t*-complex (5–40 Mb on chromosome 17). For each subspecies, we observed three distinct tree topologies (Figure 2, A–C): (1) at least one of their *t*-haplotypes was positioned within the subspecies; (2) at least one of their *t*-haplotypes was positioned within the *M. musculus* clade, but all their *t*-haplotypes were outgroups relative to the noncarriers of the subspecies; and (3) all *t*-haplotypes from the subspecies were located outside of the *M. musculus* clade. The first type of windows supports more recent and/or extensive recombination events, and the second type older or smaller-scale recombination events. We also obtained phylogenetic trees for each of these windows using maximum parsimony and neighbor-joining approaches (Figure S7, A and B in File S2, respectively). The resulting tree topologies are consistent for all three methods in 75% (*M. m. castaneus*) to 85% (*M. m. domesticus* and *M. m. musculus*) of the windows, and similarly distributed along the *t*-haplotype for all the methods.

There is a good correspondence between the peaks of high divergence and regions where most windows show no evidence of recent recombination between the *t* and standard chromosomes (green bars in Figure 1). The phylogenetic patterns along the *t*-haplotype therefore support the view of four ancient inversions, of which large sections have been replaced by genetic material from the standard chromosome, likely through occasional events of recombination between the two.

### The phylogeny of the *t*-haplotype does not mirror that of the standard chromosome, but does not support a single recent introgression

A previous phylogenetic analysis suggested that a single *t*-haplotype introgressed into all *M. musculus* subspecies < 0.8 MYA (Morita *et al.* 1992). Figure 3A shows the expected phylogeny under this scenario: all *t*-haplotypes are highly diverged from the standard chromosomes, but very similar to each other, and the *t*-haplotype tree is polytomic. Figure 3B is the expected phylogeny if *t*-haplotypes were present in the three *M. musculus* subspecies before these split, and have been maintained in each independently. In this case, the phylogenetic topology of *t*-haplotypes reflects the history of the *M. musculus* species complex.

To test these two models, we estimated the phylogeny of the 15 *t*-haplotypes and 40 noncarrier mice using only the 364 5-kb windows for which no recombination could be detected using any subspecies and phylogenetic method (to exclude signals caused by recent genetic exchange with the standard chromosomes). Figure 3C shows that the resulting phylogeny is not fully consistent with a very recent sweep of a single *t*-haplotype across the three subspecies: *t*-haplotypes have diverged sufficiently for *M. m. castaneus* and *M. m. domesticus t*-haplotypes to cluster by subspecies, while *M. m. musculus t*-haplotypes are outside of the *M. m. castaneus*/*M. m. domesticus* cluster. Since polytomies can mistakenly yield highly supported resolved trees (White *et al.* 2009), we tested whether the branch leading to the *M. m. castaneus*/*M. m. domesticus* cluster was significantly different from zero (Almeida *et al.* 2011). We took the maximum likelihood tree shown in Figure 3C, manually collapsed this branch, and ran the IQ-tree “tree topology test” on the original and the polytomic trees. This yielded much higher support for the original tree [*P* = 0, Shimodaira–Hasegawa test (Shimodaira and Hasegawa 1999)].

We ran two more controls to check that the clustering of the *M. m*. *castaneus* and *M. m. domesticus t*-haplotypes was not an artifact of the data or analysis (Figure S8 in File S2). First, we reestimated the phylogeny using more stringent pseudo-*t*-haplotype SNP profiles, which included only SNPs that were not found in any of the noncarriers of any subspecies, even if they were homozygous in *t*-carriers (Figure S8B in File S2). The *M. m. musculus t*-haplotypes remained outside of the *M. m. domesticus/M. m. castaneus* cluster. Second, we applied our SNP subtraction pipeline to the rest of chromosome 17 (50–90 Mb), to check that our procedure was removing enough SNPs from *t*-carriers to prevent them from clustering by subspecies simply due to residual variants (Figure S8A in File S2). This yielded an unresolved species tree for the *t*-carriers.

The data were also inconsistent with a simple model of maintenance of an ancestral *t*-haplotype in the three subspecies: while the species tree obtained for the noncarriers reflected the presumed history of the species complex (White *et al.* 2009), with *M. m. castaneus* and *M. m. musculus* clustering as sister species, *M. m. musculus* was an outgroup to the other two for the *t*-haplotype. This discrepancy was fairly consistent: 83% of the resolved 5-kb windows clustered *M. m. musculus* and *M. m. castaneus* for the noncarrier individuals, whereas 54% of such windows placed *M. m. musculus* as an outgroup for the *t*-haplotype (Figure 3D). Maximum parsimony and neighbor-joining approaches also supported primarily the *M. m. domesticus/M. m. castaneus t*-haplotype sister relationship (Figure S9, A and B in File S2, respectively). This confirms that *t*-haplotypes were still exchanged between the subspecies during early speciation (Morita *et al.* 1992), but suggests that some genetic flow persisted for longer between *M. m. domesticus* and *M. m. castaneus*.

While these patterns generally hold using our alternative SNP-filtering procedures (Figure S1 and Figure S2 in File S2), the allele frequency filtering yields support for both the *M. m. castaneus/M. m. domesticus* and the *M. m. musculus/M. m. domesticus* sister relationships. This seems to be driven by the loss of many heterozygous SNPs in low-coverage individuals due to deviations in allele frequency from 50%; when only a coverage filter is applied, the results once again support primarily the *M. m. castaneus/M. m. domesticus* clustering of *t*-haplotypes.

### Recombination with the standard chromosome counteracts the genetic deterioration of the *t*-haplotype

We estimated the ratio of nonsynonymous to synonymous SNPs (NS/S) of the pseudo-*t*-haplotype SNP profiles and compared it to the respective ratio for noncarrier *M. m. domesticus* individuals. When all SNPs are considered, NS/S is similar (0.74 for the *t*-haplotypes and 0.69 for noncarriers); however, most nonsynonymous SNPs found on the standard chromosomes are segregating at low frequency, as expected if they are overall deleterious, whereas many are fixed or at high frequency on the *t*-haplotype. We therefore reestimated NS/S for different SNP frequency classes (Figure 4A) among the four *t*-haplotypes, 24 control *M. m. domesticus* and 16 *M. spretus* chromosomes, for the *t*-complex region. As expected, both *M. m. domesticus* and *M. spretus* show a decreased NS/S ratio for high-frequency SNPs. Pseudo-*t*-haplotypes harbor an excess of nonsynonymous SNPs for all frequency classes. This difference is more pronounced for mutations that are shared by 50–100% of the chromosomes (*P* = 0.07 and *P* = 0.007 for frequency classes 0.75 and 1, respectively, between *M. m. domesticus* and the *t*-haplotypes, and *P* = 0.006 and *P* = 0.06 for the corresponding comparisons between *M. spretus* and the *t*-haplotype using Yates-corrected χ^2^ test), consistent with the idea that the *t*-haplotype has accumulated an excess of deleterious variants.

It was recently suggested that occasional gene flow between a meiotic drive system of *Drosophila* and the standard chromosome was sufficient to purge deleterious mutations from the driver (Pieper and Dyer 2016), and that this may contribute to its long-term viability. We similarly hypothesized that occasional recombination between the *t*-haplotype and the standard chromosome may contribute to the regeneration of coding sequences, so fixed SNPs in nonrecombined regions should have higher NS/S overall than regions that have recently recombined with the standard chromosome. We assigned SNPs to nonrecombined, recently or very recently recombined regions of the *t*-complex based on the *M. m. domesticus* phylogenetic topologies shown in Figure 2. For each category, we computed NS/S for SNPs found on the *t*-haplotypes and on *M. spretus* (as a control for differences in selective pressure along the chromosome). Figure 4B shows that while *M. spretus* harbors no difference in NS/S between the three types of regions, *t*-haplotypes have higher NS/S in the nonrecombined regions than in either class of recombined regions (*P* < 0.001, Yates-corrected χ^2^ tests) and the corresponding regions in *M. spretus* (*P* = 0.008). The two classes of recombined regions are not significantly different from each other or from the corresponding regions in *M. spretus*.

The decreased NS/S in the most recently/extensively recombined regions was observable using both of our alternative filtering procedures (Figure S1 and Figure S2 in File S2), but did not yield significant differences for the third filtering procedure (based on coverage and allele-specific frequency, Figure S2 in File S2), again due to the removal of many heterozygous SNPs in low-coverage individuals.

### CNVs drive expression divergence in *t*-haplotype carriers

While several chromosome 17 genes were found to differ in expression between *t*-haplotype carriers and noncarriers (Lader *et al.* 1989; Ha *et al.* 1991; Braidotti and Barlow 1997; Zwart *et al.* 2001), the overall effect of carrying a degenerating *t*-haplotype on genome-wide patterns of gene expression has not yet been assessed. We took advantage of the availability of RNA-seq data for several tissues derived from the same *M. m. domesticus* invidividuals (Harr *et al.* 2016) to contrast gene expression levels (in Transcripts Per Million, TPM) between the four *t*-carrier mice and all noncarriers from France and Germany (Table S1 in File S2).

**Figure 5:**
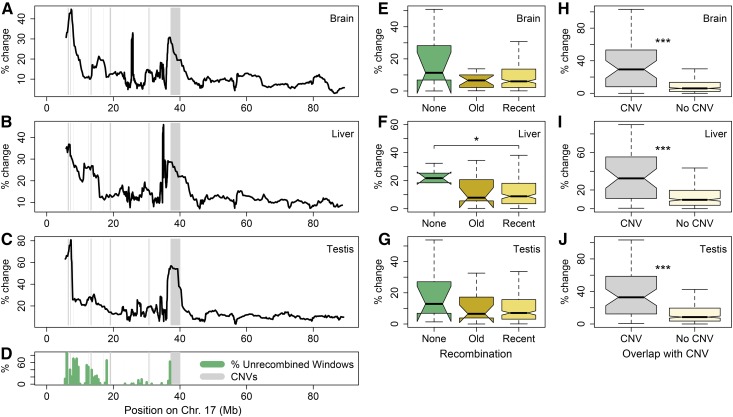
Divergence of gene expression between *t*-carriers and noncarriers. (A–C) Percentage difference between the average gene expression of *t*-carrier and noncarriers (estimated as: | average_t-carrier − average_noncarrier | / average_noncarrier), plotted using a sliding window of 20 genes (using all genes with expression values > 10 in noncarriers). Expression divergence is shown for (A) the brain, (B) the liver, and (C) the testis. Regions that contain *t*-specific copy number variants (CNVs) (obtained by comparing the coverage of *t*-carriers to noncarriers, see *Materials and Methods*) are marked by gray rectangles. (D) The percentage of 5-kb windows for which no recombination was detected on *M. m. domesticus* t-haplotypes. (E–G) Boxplots showing the percentage difference in expression of *t*-carriers relative to that of noncarriers for genes that overlap with at ≥ 80% 5-kb windows for which no recombination was detected (green), some/old recombination was detected (orange), and recent/extensive recombination was detected (yellow), in (E) the brain, (F) the liver, and (G) the testis. (H–J) Boxplots showing the percentage difference in expression of *t*-carriers relative to that of noncarriers for genes overlapping or not overlapping a CNV, in (H) the brain, (I) the liver, and (J) the testis.

Figure 5 shows the extent to which expression has changed along the *t*-haplotype in brain, liver, and testis (other tissues are shown in Figure S10 in File S2), using a sliding window of 20 genes. Although different genes are differentially expressed in each tissue (Table S1 in File S2 and supplemental data in File S2), the general patterns of divergence are similar for all tissues, with large peaks of expression divergence at ∼5 and 39 Mb. Both of these regions overlap with CNVs that were detected when the coverage of *t*-haplotype carriers and noncarriers was compared (gray bars in Figure 5).

Duplications and deletions are known to affect gene expression and have been detected for several differentially expressed genes on the *t*-haplotype (Braidotti and Barlow 1997; Zwart *et al.* 2001). Similarly, gene amplification was recently found to be essential for R2D2, another mouse meiotic driver (Didion *et al.* 2016; Morgan *et al.* 2016), and meiotic drivers on the mouse sex chromosomes have been postulated to lead to the extensive gene amplification that is observed on both the X and Y chromosomes (Soh *et al.* 2014). We therefore tested whether genes that overlapped with *t*-specific CNVs had diverged more in expression than the rest of the *t*-haplotype. The boxplots in Figure 5 show that this is indeed the case (*P* < 10^−6^ in all three tissues), with a median change of > 30% for CNV-overlapping genes *vs.* 10% for other genes.

Genes located in windows that have recently recombined with the standard chromosome were expected to show lower levels of gene expression divergence. We classified genes into each recombination class if ≥ 80% of the windows they overlapped with were of that class. While genes in recently recombined regions had a lower median percentage change than unrecombined windows in all tissues (Figure 5 and Figure S10 in File S2), the difference was generally not significant. This is likely due to the fact that highly diverged regions often overlap with CNVs (Figure 1) and were excluded from our phylogenetic analysis, such that only a few genes were left in unrecombined regions.

Finally, while our differential expression analysis in the testis recovered mainly chromosome 17 genes that were previously known to differ in expression in *t*-carriers (Lader *et al.* 1989; Ha *et al.* 1991; Braidotti and Barlow 1997; Zwart *et al.* 2001), one of the genes with the lowest *q*-value, Ppp1cb, is located on chromosome 5; despite not being on the *t*-haplotype, it shows a consistent 10-fold overexpression in *t*-carriers (Table S1 in File S2). Protein phosphatase 1 proteins are known to be essential for spermatogenesis (Silva *et al.* 2014). One of the active forms of PP1 has been shown to repress sperm motility in the epididymis, making Ppp1cb a promising candidate for involvement in drive and/or response to the driver (Vijayaraghavan *et al.* 1996). Another 2 out of 12 differentially expressed genes (Dr1 and Scamp2) are located on other chromosomes, emphasizing that regulatory changes on the *t*-haplotype can affect its biology through changes in the expression of genes located on other chromosomes.

## Discussion

Despite having been studied for close to a century, reduced recombination rates on the *t*-haplotype have limited the power of traditional genetic studies for this selfish element, and next-generation sequencing approaches offer a promising alternative to complement this body of work.

The variable levels of divergence along the *t*-haplotype complicate the inference of the history of the four inversions. While large sections of inversion 4 have lower divergence levels than the other inversions, as expected if it was acquired later (Hammer and Silver 1993), a peak of very high divergence is found at ∼37–40 Mb. Two hypotheses could account for this: (1) inversion 4 may be of similar age as inversion 2, but much of its differentiation may have been lost through recombination with the standard chromosome, and (2) this region may have particularly high rates of divergence. Although we do find evidence of recombination over much of inversion 4, the region of highest divergence contains clusters of olfactory, immune, and pheromone genes, all of which tend to be highly polymorphic and fast evolving (Figure S3 in File S2). We control for these by normalizing by the noncarrier heterozygosity, by checking that the pattern holds when only neutral SNPs are used (Figure S4B in File S2), and by comparing the SNP density of the pseudo-*t*-haplotype to the SNP density of *M. spretus* (Figure S4, C–F in File S2). However, the reduced recombination rates on the *t*-haplotype may have led to the fixation of many ancestral neutral polymorphisms, and consequently increased rates of neutral divergence specifically on the *t*-haplotype. These results are therefore not incompatible with a younger age of inversion 4, and emphasize that care should be taken when interpreting data obtained from small genomic regions.

Genetic exchange between the *t*-haplotype and the standard homolog was supported both by the phylogenetic topology and by the colocalization of some of the most diverged regions with putative inversion boundaries. Although several studies have found evidence for small-scale gene conversion in the fourth and largest inversion (Herrmann *et al.* 1987; Erhart *et al.* 1989, 2002; Hammer *et al.* 1991; Wallace and Erhart 2008), the extent of recombination that we observe here is unexpected, as repressed recombination is thought to be a hallmark of successful segregation distorters. However, it is in-line with Pieper and Dyer (2016), who suggested that occasional recombination events could provide a mechanism to counteract the accumulation of deleterious mutations on meiotic drivers due to Hill–Robertson effects. Consistent with this, the excess of fixed nonsynonymous SNPs on the *t*-haplotype is reduced in regions for which we detect recent recombination. Another effect of occasional gene flow with the standard homolog may be the maintenance of optimal gene expression levels on the *t*-haplotype. Although several genes showed altered expression in *t*-carriers (12 out of 463 genes in the testis, fewer in other tissues), the vast majority did not, and it is likely that such conserved expression results at least in part from genetic homogenization due to recombination.

Finally, our phylogenetic analysis uncovered variation between *t*-haplotypes sampled from the three *M. musculus* subspecies, and a phylogenetic topology that disagrees with that of the standard subspecies tree, but also seems inconsistent with a very recent introgression of a single *t*-haplotype. Some caveats should be taken into account when interpreting these data. First, we use pseudo-*t*-haplotypes, which may contain some residual SNPs from the standard chromosome, and it will be important to confirm these results using sequences derived from homozygous *t*-carriers. Second, gene conversion from the standard chromosome to the *t*-haplotype could result in *t*-haplotypes becoming quickly differentiated after introgressing. Finally, the evolutionary history of the *M. musculus* subspecies complex is itself challenging to disentangle, as the three subspecies are estimated to have diverged less than half a million years ago, and because there is a varying rate of gene flow between them and across genomic regions (Geraldes *et al.* 2008). Despite this variance, 39% of the genome supports the *M. m. musculus/M. m. castaneus* sister species relationship (White *et al.* 2009), which is most likely the primary phylogenetic history (in agreement with our findings for noncarriers). Contrary to this, the *M. m. castaneus* and *M. m. domesticus t*-haplotypes are most closely related in 53% of the windows. However, the fact that the other two topologies are also supported by 16% (for the *M. m. musculus/M. m. castaneus* cluster) and 30% (*M. m. domesticus/M. m. castaneus* cluster) of windows suggests that, similar to what occurred on the standard chromosome, gene flow between the *t*-haplotypes of the different subspecies may have shaped the phylogenetic topology of this large meiotic driver, as expected if *t*-haplotypes were being regularly exchanged between subspecies during early speciation.

### Conclusions

Our global analysis of the sequence and expression patterns of the *t*-haplotype confirmed its ancient origin, the involvement of large parts of chromosome 17, and revealed an excess of nonsynonymous mutations consistent with the genetic deterioration that is expected in the absence of recombination. Surprisingly, this was counteracted by occasional recombination with the standard chromosome over a large proportion of the *t*-complex, providing an explanation for its long-term survival. Finally, the fact that most of the change in gene expression is driven by the accumulation of CNVs, but that regulatory changes on the *t*-haplotype can also affect the expression of genes elsewhere, provides new insights into the biology of the *t*-haplotype, and opens new avenues of exploration for this model segregation distorter.

## 

## Supplementary Material

Supplemental material is available online at www.genetics.org/lookup/suppl/doi:10.1534/genetics.117.300513/-/DC1.
