# Supplementary material for: Complex History and Differentiation Patterns of the t-Haplotype, a Mouse Meiotic Driver
Source: Genetics. 2017 Nov 14;208(1):365–75. doi: 10.1534/genetics.117.300513 (PMC5753869; doi:10.1534/genetics.117.300513)
Supplement: Supplementary file 1 [file 365FileS1.pdf]

File S1: Supplementary Methods

Contents

1 Summary of mouse samples **2**

2 CNV detection **5**
2.1 Control individuals 5
2.2 *t*-haplotype carriers 5
2.3 Control-FREEC 6
2.4 Merging list of CNV regions found by Control-FREEC and reported by Harr *et al.* 8

3 Filtering of VCF files **8**
3.1 SNP filtering procedure 1: High quality (PASS) SNPs 9
3.2 Removing CNV regions from VCF files 9

4 Estimating divergence **9**

5 Estimating trees in 5 Kb windows **10**
5.1 Extracting pseudo-*t*-haplotypes from *t*-carriers 10
5.2 Running a phylogenetic software on the FASTA files in windows of 5 Kb 11

6 Inferring topology from trees in non-recombined regions of the *t*-haplotype **12**
6.1 Estimating tree based only on 5 Kb windows that did not recombine in any of the subspecies 12

7 Deterioration **14**

8 Expression analysis **15**
8.1 Differential expression analysis 15

9 Controls using SNP filtering 2 and 3 **16**
9.1 SNP filtering procedure 2: Coverage filtering using sample-specific overall coverage cutoff 16
9.2 SNP filtering procedure 3: Coverage filtering using sample-specific overall coverage cutoff, and cutoff for allele coverage in heterozygous sites 16

10 Controls for divergence **17**
10.1 Neutral divergence 17
10.2 Divergence of the pseudo-t-haplotypes normalized by *Mus spretus* 17

11 Controls for inferring topology from trees in non-recombined regions of the t-haplotype **19**

Supplementary Code

We have created a folder that contains additional scripts and configuration files, some of which we referred to in this document. You can find this folder here: `http://dx.doi.org/10.15479/AT:ISTA:79`.

Reference genome

Throughout our analysis, we used the published house mouse reference genome, version *mm10*. `https://www.ncbi.nlm.nih.gov/grc/mouse`

1 Summary of mouse samples

We used data from 55 mice published by Harr *et al.* DOM stands for the genus *Mus musculus domesticus*, MUS for *Mus musculus musculus*, CAS for *Mus musculus castaneus* and SPRET for *Mus spretus*.

Subspecies	Origin	Sex	Mouse ID	t-carrier
DOM	France	male	14	No
DOM	France	male	15B	No
DOM	France	male	16B	No
DOM	France	male	18B	No
DOM	France	male	B2C	Yes
DOM	France	male	C1	Yes
DOM	France	male	E1	No
DOM	France	male	F1B	Yes
DOM	Germany	male	TP1	No
DOM	Germany	male	TP121B	No
DOM	Germany	male	TP17-2	No
DOM	Germany	male	TP3-92	No
DOM	Germany	male	TP4a	No
DOM	Germany	male	TP51D	Yes
DOM	Germany	male	TP7-10	No
DOM	Germany	male	TP81B	No

Subspecies	Origin	Sex	Mouse ID	t-carrier
MUS	Afghanistan	male	396	Yes
MUS	Afghanistan	male	413	No
MUS	Afghanistan	male	416	Yes
MUS	Afghanistan	male	424	No
MUS	Afghanistan	female	435	No
MUS	Afghanistan	male	444	Yes
MUS	Czech Republic	female	CR12	Yes
MUS	Czech Republic	female	CR13	Yes
MUS	Czech Republic	male	CR16	No
MUS	Czech Republic	female	CR23	Yes
MUS	Czech Republic	male	CR25	No
MUS	Czech Republic	male	CR46	No
MUS	Czech Republic	female	CR59	No
MUS	Kazakhstan	female	AL1	No
MUS	Kazakhstan	male	AL16	No
MUS	Kazakhstan	female	AL19	Yes
MUS	Kazakhstan	female	AL33	No
MUS	Kazakhstan	male	AL38	No
MUS	Kazakhstan	female	AL40	No
MUS	Kazakhstan	male	AL41	Yes
MUS	Kazakhstan	male	AL42	No
CAS	India	male	H12	Yes
CAS	India	female	H14	Yes
CAS	India	female	H15	No
CAS	India	female	H24	No
CAS	India	female	H26	No
CAS	India	female	H27	Yes
CAS	India	male	H28	No
CAS	India	female	H30	No
CAS	India	male	H34	No
CAS	India	female	H36	No

Subspecies	Origin	Sex	Mouse ID	t-carrier
SPRET	Spain	female	SP36	No
SPRET	Spain	male	SP39	No
SPRET	Spain	male	SP41	No
SPRET	Spain	female	SP51	No
SPRET	Spain	female	SP62	No
SPRET	Spain	male	SP68	No
SPRET	Spain	male	SP69	No
SPRET	Spain	male	SP70	No

2 CNV detection

2.1 Control individuals

We randomly selected the following non-t-haplotype-carrier individuals to be controls in detecting t-haplotype-specific copy number variants:

Subspecies	Population	Mouse ID
DOM	France	14
DOM	France	18B
DOM	Germany	TP1
DOM	Germany	TP4
MUS	Afghanistan	424
MUS	Afghanistan	435
MUS	Czech Republic	CR16
MUS	Kazakhstan	AL1
CAS	India	H15
CAS	India	H26
CAS	India	H30
CAS	India	H36

2.2 *t*-haplotype carriers

The following individuals carry the t-haplotype (CR29 from the Czech Republic was excluded from all analysis because its SNP density profile suggested that it is a partial t-haplotype carrier)

Subspecies	Population	Mouse ID
DOM	France	C1
DOM	France	B2C
DOM	France	F1B
DOM	Germany	TP51
MUS	Afghanistan	396
MUS	Afghanistan	416
MUS	Afghanistan	444
MUS	Czech Republic	CR12
MUS	Czech Republic	CR13
MUS	Czech Republic	CR23
MUS	Kazakhstan	AL19
MUS	Kazakhstan	AL41
CAS	India	H12
CAS	India	H14
CAS	India	H27

2.3 Control-FREEC

We used the software Control-FREEC to identify copy number variants. For the *t*-haplotype-carriers we either called CNVs relative to the house mouse reference, or to a control sample. For all non- *t*-haplotype-carriers we called CNVs relative to the reference genome. For all *t*-haplotype-carriers and non-*t*-haplotype-carriers, and in calls relative to a control sample or the reference, we used two different window size settings, 5 Kb or 1 Kb.

Calling CNVs using the reference genome:

```
freec -conf config_B2C_ref.text
```

The configuration file config_B2C_ref.txt contained the following lines, where *window* was either 5000 or 1000.

```
[general]

chrLenFile = Mus_musculus_wholeGenome_reference_versionGRCm38_chrInSeqName.fa.fai
ploidy = 2
window=5000
chrFiles = chr_files/

[sample]
```

```
mateFile = B2C_sorted.cigar.nodups.realigned.recalibrated.sorted.bam
inputFormat = BAM
mateOrientation = 0
```

Calling CNVs using a control sample involved merging the BAM files from four non-*t*-carrier mice, and then supplying the control sample in the configuration file for Control-FREEC.

```
samtools merge Controls_DOM.bam
14_sorted.cigar.nodups.realigned.recalibrated.sorted.bam
18B_sorted.cigar.nodups.realigned.recalibrated.sorted.bam
TP1_sorted.cigar.nodups.realigned.recalibrated.sorted.bam
TP4a_sorted.cigar.nodups.realigned.recalibrated.sorted.bam

samtools index Controls_DOM.bam

freec -conf config_B2C.text
```

The configuration file:

```
[general]

chrLenFile = Mus_musculus_wholeGenome_reference_versionGRCm38_chrInSeqName.fa.fai
ploidy = 2
window=1000

[sample]

mateFile = B2C_sorted.cigar.nodups.realigned.recalibrated.sorted.bam
inputFormat = BAM
mateOrientation = 0

[control]

mateFile = Controls_DOM.bam
inputFormat = BAM
mateOrientation = 0
```

2.4 Merging list of CNV regions found by Control-FREEC and reported by Harr *et al.*

We concatenated the files produced by Control-FREEC for all the mice and extracted the first three columns to create a BED format. Since the output of this software printed only "1" for "chr1", we added the prefix "chr" before each entry of the chromosome column.

```
cat *_CNVs | awk -v OFS='\t' '{print $1, $2, $3}' > All.cnvs.bed
awk '{$1="chr"$1} 1' All.cnvs.bed > All.cnvs.chrAdded.bed
```

To combine the above file with the CNV lists provided by Harr *et al.*, we removed the first line of each file, because those did not contain relevant data. We printed tab-delimited files for each subspecies and *M. spretus*.

```
awk -v OFS='\t' 'NR>1 {print $1,$2,$3}' CNV_CAST.bed >
 CNV_CAST_no1stRow.bed
```

Then we concatenated the four files with the list of CNV regions given by Control-FREEC.

```
cat *bed > Combined.cnvs.bed
```

We sorted the BED file on the first and on the second column, and merged the CNV region using BEDtools' *merge* function. This way we got rid of duplicates and obtained a list of regions that have been found at least once to vary in copy number relative to the wild type or to a group of control individuals.

```
sort -k 1,1 -k 2n,2n Combined.cnvs.bed > Combined.cnvs.sorted.bed
~/Software/bedtools2/bin/bedtools merge -i Combined.cnvs.sorted.bed >
CNVRegions.bed
```

3 Filtering of VCF files

We downloaded the VCF files containing variant positions for all mice in all populations, provided by Harr *et al.*. Two files were provided, one with the highest-quality variants given by GATK's VariantRecalibrator tool, containing SNPs and MNPs, and one with all raw variants given by GATK's HaplotypeCaller tool, containing INDELs, SNPs and MNPs. This file of raw variants we used to conduct our own coverage-based filtering on, and which then served as an alternative list of variants to test the robustness

of our downstream analyses and results. When manipulating VCF files we used the software BCFtools, version 1.3. Here we show the analysis using high quality SNPs (SNP filtering procedure 1), and in Section 8 we highlight any changes in the filtering pipeline when using the raw variant list (SNP filtering procedure 2 and 3).

3.1 SNP filtering procedure 1: High quality (PASS) SNPs

We downloaded the file containing the high quality PASS-labeled SNPs. This filtering procedure only involved the removal of any variant that was not labeled a SNP.

```
wget http://wwwuser.gwdg.de/~evolbio/evolgen/wildmouse/vcf/
AllMouse.vcf_90_recalibrated_snps_raw_indels_reheader_PopSorted.PASS.vcf.gz
```

Once downloaded we excluded from this file all variant sites where at least one of the alternative alleles was an MNP.

```
~/Software/bcftools-1.3/bcftools view -V mnps  -O z AllMouse_PASS.vcf.gz >
AllMouse_PASS_snps.vcf.gz
```

3.2 Removing CNV regions from VCF files

Subsequently, we removed all regions identified as CNVs from both files.

```
~/Software/bcftools-1.3/bcftools view -T ^CNVRegions.bed -O z
AllMouse_PASS_snps.vcf.gz > AllMouse_PASS_snps_maskedCNVs.vcf.gz
```

4 Estimating divergence

As a measure of divergence we used heterozygosity of t-carriers normalized by the heterozygosity of non-t-carriers. We used the complete SNP list for this analysis, without removing CNV regions, and instead we indicated the abundance of CNVs in the plot.

```
for sample in GER1_TP1.variant2 Mmd_GER2_TP121B.variant2 ...;
do
~/Software/bcftools-1.3/bcftools view -s $(echo ${sample})
```

```
AllMouse_PASS_snps.vcf.gz  > temp.vcf
~/Software/bcftools-1.3/bcftools view -H -e'GT="0/0"|GT="."|GT="1/1"
|GT="2/2"|GT="3/3"' temp.vcf | awk '{print $2}'
> $(echo ${sample}.het.coordinates);
done
```

Then we used the R script DIVERGENCE.R (see Supplementary Code) to compute average SNP densities in non-t and t-carriers in 100bp bins, and plot the ratio of sliding window averages in 1 Mb windows.

5 Estimating trees in 5 Kb windows

5.1 Extracting pseudo-t-haplotypes from t-carriers

We used the following pipeline to retain SNPs in t-carriers that are either homozygous, or heterozygous, and not observed in any non-t-carrier individual.

```
inFile=AllMouse_PASS_snps_maskedCNVs_Tcomplex.vcf.gz

WTs=Mmm_CZE3_CR16.variant5,Mmm_CZE5_CR25.variant5...

for t in Mmd_GER6_TP51D.variant2 Mmd_FRA6_C1.variant ... ;
 do
~/Software/bcftools-1.3/bcftools view -O z -s $(echo ${t},${WTs})
$inFile > tempSamps.vcf.gz

~/Software/bcftools-1.3/bcftools view -O z --private -s $(echo ${t})
tempSamps.vcf.gz > tempPriv.vcf.gz

~/Software/bcftools-1.3/bcftools view -O z -e'GT="1/1" | GT="2/2"
 | GT="3/3" | GT="."' tempPriv.vcf.gz > tempPrivHET.vcf.gz

~/Software/bcftools-1.3/bcftools view -O z -s $(echo ${t}) $inFile
 > tempSamp.vcf.gz
~/Software/bcftools-1.3/bcftools view -O z -i'GT="1/1" | GT="2/2" |
GT="3/3"' tempSamp.vcf.gz > tempHOM.vcf.gz

~/Software/bcftools-1.3/bcftools index tempPrivHET.vcf.gz
~/Software/bcftools-1.3/bcftools index tempHOM.vcf.gz
```

```
~/Software/bcftools-1.3/bcftools concat -a -D -o sample_tHaplSubset.vcf.gz
-O z tempHOM.vcf.gz tempPrivHET.vcf.gz

~/Software/bcftools-1.3/bcftools index sample_tHaplSubset.vcf.gz
```

The following command was used to convert the list of variants for pseudo-t-haplotypes and non-t-carriers to FASTA files:

```
~/Software/bcftools-1.3/bcftools consensus -H 2 -f reference.fa
 -o sample.fa sample.vcf.gz
```

5.2 Running a phylogenetic software on the FASTA files in windows of 5 Kb

We concatenated all the FASTA files into one multisample FASTA file called CompleteMice.fa. Then we took non-overlapping 5Kb regions along the t complex' region (chr17:5-40Mb) and called the maximum likelihood estimation software IQTree on the subset region's FASTA file.

```
for i in ‘seq 1 7000‘; do
inFile=CompleteMice.fa #input file

Reg=$(echo $[0+($i-1)*5000]-$[0+$i*5000])

headers=$(awk '{print $1}' $(echo ${inFile}.fai))

~/Software/samtools-1.3/samtools faidx $(echo ${inFile})
$(echo $(for header in ${headers};do echo -n "$header:$Reg "; done))
 > $(echo ${Reg}.fa) #subset fasta file

~/Software/iqtree-omp-1.4.2-Linux/bin/iqtree-omp -nt 8 -m HKY
-bb 1000 -s $(echo ${Reg}.fa)
```

Calls to alternative phylogenetic software were the following. Using the software MEGA, we kept the default parameters:

```
for file in FASTA/*
do
/Users/Shared/megacc -a infer_single_MP_nucleotide.mao -d ${file} -o ${file}.mp
done
```

FastPhylo:

```
for file in FASTA/*
do
cat ${file} | perl -pi -e 's/\//_/gi' |
~/fastphylo-1.0.1-Linux/bin/fastdist |
~/fastphylo-1.0.1-Linux/bin/fnj -O newick > ${file}.nj
done
```

We inferred whether a given subspecies' pseudo-t-haplotypes were all outside of the *Mus musculus* species complex, inside of it, or inside even their own subspecies clade. To do this we parsed the tree file of each 5 Kb window by a following Perl script named TreeParser_SubspeciesNameHere.pl. Then we used the following wrapper code to call this on all trees:

```
for file in Trees/*

do
perl TreeParser\_SubspeciesNameHere.pl ${file}
rm ${file}.simple

done
```

6 Inferring topology from trees in non-recombined regions of the t-haplotype

6.1 Estimating tree based only on 5 Kb windows that did not recombine in any of the subspecies

We first created a BED file with the start and end coordinates of the non-recombined 5 Kb windows. Coordinates are not real genomic coordinates, but the coordinates in the 35 Mb region of the t complex. We set the start and end coordinates of the t complex to be 5 Mb and 40 Mb of chromosome 17.

```
tcomplex        2445000 2450000
tcomplex        2460000 2465000
tcomplex        2530000 2535000
tcomplex        2535000 2540000
tcomplex        2565000 2570000
tcomplex        3170000 3175000
tcomplex        3180000 3185000
```

We wanted to create a complementary BED file, which contains the regions to be masked at a subsequent step. We created a .genome file for BEDTools to use when complementing the non-recombined regions. This file contains the sequence ID, "t complex", and the length of the sequence, which is 35 Mb in our case.

```
tcomplex        35000000
```

Then we used BEDtools to create a complementary BED file.

```
bedtools complement -i NonRecombWindows_inAll3Subsepcies_AllMethods.bed
 -g t_complex.genome  | awk 'OFS="\t" {print $2,$3}'  > recombWindows
```

```
tcomplex        0       2445000
tcomplex        2450000 2460000
tcomplex        2465000 2530000
tcomplex        2540000 2565000
tcomplex        2570000 3170000
tcomplex        3175000 3180000
```

Then we create a BED file with each mouse sample and recombined window coordinates, to mask these regions in all mice in the combined FASTA file.

```
for s in $(awk '{print $1}' CompleteMice.fa.fai);
do
while read window; do echo -e "${s}\t"$window;
done < recombWindows;done |
awk 'OFS="\t" {print $1,$2,$3}'  > MaskFasta.bed
```

We then ran BEDtools' *maskfasta* command to replace every base in the recombined regions of the combined FASTA file CompleMice.fa with Ns.

```
bedtools maskfasta -fi CompleteMice.fa -bed
MaskFasta.bed -fo CompleteMiceMasked.fa
```

Then we estimated phylogeny using the software IQTree:

```
~/Software/iqtree-omp-1.4.2-Linux/bin/iqtree-omp -nt 8
-m HKY -bb 1000 -s CompleteMiceMasked.fa
```

7 Deterioration

We annotated the T complex (chr17:5-40Mb) VCF file using the software SnpEff and then extracted the coordinates for homozygous and heterozygous missense and synonymous variants for each sample.

For non-*t*-carriers:

```
java -Xmx4g -jar ~/Software/snpEff/snpEff.jar GRCm38.82
 AllMouse_PASS_snps_maskedCNVs_Tcomplex.vcf.gz >
 AllMouse_PASS_snps_maskedCNVs_Tcomplex.vcf.gz.chr17.ann

 inFile=AllMouse_PASS_snps_maskedCNVs_Tcomplex.vcf.gz.chr17.ann

for sample in Mmd_GER1_TP1.variant2 Mmd_GER2_TP121B.variant2 ... ;
do
~/Software/bcftools-1.3/bcftools view -s $(echo ${sample}) $inFile > temp.vcf

~/Software/bcftools-1.3/bcftools view -H -i'GT="1/1"|GT="2/2"|GT="3/3"'
temp.vcf | awk '/missense_variant/ {print $2}' >
 $(echo ${sample}.hom.missense)

~/Software/bcftools-1.3/bcftools view -H -i'GT="1/1"|GT="2/2"|GT="3/3"'
temp.vcf | awk '/synonymous_variant/ {print $2}' >
 $(echo ${sample}.hom.synonymous)

~/Software/bcftools-1.3/bcftools view -H -i'GT="0/1"|GT="0/2"|GT="0/3"
|GT="1/2"|GT="2/3"|GT="1/3"' temp.vcf | awk '/missense_variant/ {print $2}'
> $(echo ${sample}.het.missense)

~/Software/bcftools-1.3/bcftools view -H -i'GT="0/1"|GT="0/2"|GT="0/3"|
GT="1/2"|GT="2/3"|GT="1/3"' temp.vcf | awk '/synonymous_variant/ {print $2}'
> $(echo ${sample}.het.synoynmous);
done
```

In the case of *t*-carriers we first extracted pseudo-*t* SNPs and then filtered those in a similar way to the above procedure.

```
WTs=Mmm_CZE3_CR16.variant5,Mmm_CZE5_CR25.variant5...

for t in Mmd_FRA5_B2C.variant Mmd_FRA6_C1.variant
Mmd_FRA8_F1B.variant Mmd_GER6_TP51D.variant2;
```

```
do
~/Software/bcftools-1.3/bcftools view -O z -s $(echo ${t},${WTs}) $inFile
 > tempSamps.vcf.gz

~/Software/bcftools-1.3/bcftools view -O z --private -s $(echo ${t})
tempSamps.vcf.gz > tempPriv.vcf.gz

~/Software/bcftools-1.3/bcftools view -O z -e'GT="1/1" | GT="2/2" |
GT="3/3" | GT="."' tempPriv.vcf.gz > tempPrivHET.vcf.gz

~/Software/bcftools-1.3/bcftools view -O z -s $(echo ${t}) $inFile
 > tempSamp.vcf.gz
~/Software/bcftools-1.3/bcftools view -O z -i'GT="1/1" | GT="2/2" |
GT="3/3"' tempSamp.vcf.gz > tempHOM.vcf.gz

~/Software/bcftools-1.3/bcftools index tempPrivHET.vcf.gz
~/Software/bcftools-1.3/bcftools index tempHOM.vcf.gz

~/Software/bcftools-1.3/bcftools concat -a -D -o temp_tHaplSubset.vcf
tempHOM.vcf.gz tempPrivHET.vcf.gz

~/Software/bcftools-1.3/bcftools view -H temp_tHaplSubset.vcf |
awk '/missense_variant/ {print $2}' > $(echo ${t}.PSEUDO.missense)
~/Software/bcftools-1.3/bcftools view -H temp_tHaplSubset.vcf |
 awk '/synonymous_variant/ {print $2}' > $(echo ${t}.PSEUDO.synonymous);
done
```

Following that we used the R script DETERIORATION.R to calculate the nonsynonymous over synonymous SNP ratio in categories of the site frequency spectra for each clade. Please refer to the Supplementary Code.

8 Expression analysis

8.1 Differential expression analysis

We used the software Kallisto for mapping transcriptomic reads to the coding sequences of the reference genome, and for quantifying transcript abundances.

```
kallisto index --index=Mouse_CDS_index Mus_musculus.GRCm38.cds.all.fa
```

```
kallisto quant -i Mouse_CDS_index -o OutputDir -b 100
sample1_1.fastq sample1_2.fastq
```

Then we used the R package Sleuth to quantify and analyze the differential expression of each transcript between non-t and t-carriers. For scripts please refer to the Supplementary Code.

9 Controls using SNP filtering 2 and 3

We downloaded the file containing raw variants and retained only SNPs in the VCF file:

```
wget http://wwwuser.gwdg.de/~evolbio/evolgen/wildmouse/vcf/
AllMouse.vcf_90_recalibrated_snps_raw_indels_reheader_PopSorted.vcf.gz

~/Software/bcftools-1.3/bcftools view -V indels,mnps -O z AllMouse_raw.vcf.gz >
AllMouse_raw_snps.vcf.gz

~/Software/bcftools-1.3/bcftools view -T ^CNVRegions.bed -O z
AllMouse_raw_snps.vcf.gz > AllMouse_raw_snps_maskedCNVs.vcf.gz
```

9.1 SNP filtering procedure 2: Coverage filtering using sample-specific overall coverage cutoff

We filtered each sample's VCF file according to coverage. We kept a variant site only if its coverage was at least half of the average coverage for that given sample. The average coverage information we downloaded from Table 1 of Harr *et al.*. We used the following command to filter the sample VCF files:

```
~/Software/bcftools-1.3/bcftools view -O z -i 'FMT/DP > 11'  Sample.vcf >
 Sample_OverallCovFiltered.vcf.gz
```

9.2 SNP filtering procedure 3: Coverage filtering using sample-specific overall coverage cutoff, and cutoff for allele coverage in heterozygous sites

We filtered each sample's VCF file according to coverage. We kept a variant site only if its coverage was at least half of the average coverage for that given sample, and if heterozygous, then each allele had to have at least 30% of the reads supporting it.

```
~/Software/bcftools-1.3/bcftools view -O z -i'((GT="1/1"|GT="2/2"|GT="3/3")
& FMT/DP > 11) | (FMT/DP > 11 & (((FMT/AD[1] > (0.3 * (FMT/DP)))
& (FMT/AD[0] > (0.3 * (FMT/DP)))) | ((FMT/AD[1] > (0.3 * (FMT/DP)))
 & (FMT/AD[2] > (0.3 * (FMT/DP)))) | ((FMT/AD[2] > (0.3 * (FMT/DP)))
 & (FMT/AD[0] > (0.3 * (FMT/DP)))) ))'  Sample.vcf >
 Sample_CovFiltered.vcf.gz
```

Further analysis of these alternatively filtered variant lists were the same as for the PASS labeled variants.

10 Controls for divergence

10.1 Neutral divergence

We re-plotted the normalized divergence of the *t*-carriers using only neutral SNPs.

We annotated the VCF file using the software SNPeff and extracted synonymous variants and variants from intergenic regions.

```
~/Software/bcftools-1.3/bcftools view -O z -r chr17 AllMouse_PASS_snps.vcf.gz
> temp.vcf.gz

java -Xmx4g -jar ~/Software/snpEff/snpEff.jar GRCm38.82 temp.vcf.gz >
AllMouse_PASS_snps.vcf.gz.chr17.ann

for sample in Mmd_GER1_TP1.variant2 Mmd_GER2_TP121B.variant2 ... ;
do
~/Software/bcftools-1.3/bcftools view -s $(echo ${sample})
AllMouse_PASS_snps.vcf.gz.chr17.ann > temp.vcf

~/Software/bcftools-1.3/bcftools view -H -e'GT="0/0"|GT="."|GT="1/1"
|GT="2/2"|GT="3/3"' temp.vcf | awk '/synonymous_variant/||(/intergenic_region/
&&!/protein_coding/) {print $2}' > $(echo ${sample}.het.neutral.coordinates);

done
```

10.2 Divergence of the pseudo-*t*-haplotypes normalized by *Mus spretus*

We used the annotated file above, and extracted pseudo-*t* and *Mus spretus* SNPs. We also subset this set to only the neutral SNPs.

For *Mus spretus*:

```
for sample in Ms_SPRE1_SP36.variant9 Ms_SPRE2_SP39.variant9 ...;
do
~/Software/bcftools-1.3/bcftools view -s $(echo ${sample})
 AllMouse_PASS_snps.vcf.gz.chr17.ann > temp.vcf

~/Software/bcftools-1.3/bcftools view -H -e'GT="0/0"|GT="."' temp.vcf
| awk '{print $2}' > $(echo ${sample}.coordinates)

~/Software/bcftools-1.3/bcftools view -H -e'GT="0/0"|GT="."' temp.vcf
| awk '/synonymous_variant/||(/intergenic_region/&&!/protein_coding/)
{print $2}' > $(echo ${sample}.neutral.coordinates);

done
```

For the *t*-carriers:

```
WTs=Mmm_CZE3_CR16.variant5,Mmm_CZE5_CR25.variant5 ...

for t in Mmd_FRA5_B2C.variant Mmd_FRA6_C1.variant
Mmd_FRA8_F1B.variant Mmd_GER6_TP51D.variant2;
do
~/Software/bcftools-1.3/bcftools view -O z -s $(echo ${t},${WTs})
AllMouse_PASS_snps.vcf.gz.chr17.ann > tempSamps.vcf.gz

~/Software/bcftools-1.3/bcftools view -O z --private -s $(echo ${t})
tempSamps.vcf.gz > tempPriv.vcf.gz

~/Software/bcftools-1.3/bcftools view -O z -e'GT="1/1" | GT="2/2" |
GT="3/3" | GT="."' tempPriv.vcf.gz > tempPrivHET.vcf.gz

~/Software/bcftools-1.3/bcftools view -O z -s $(echo ${t})
AllMouse_PASS_snps.vcf.gz.chr17.ann > tempSamp.vcf.gz

~/Software/bcftools-1.3/bcftools view -O z -i'GT="1/1" | GT="2/2" |
GT="3/3"' tempSamp.vcf.gz > tempHOM.vcf.gz

~/Software/bcftools-1.3/bcftools index tempPrivHET.vcf.gz
~/Software/bcftools-1.3/bcftools index tempHOM.vcf.gz
```

```
~/Software/bcftools-1.3/bcftools concat -a -D -o temp_tHaplSubset.vcf
tempHOM.vcf.gz tempPrivHET.vcf.gz

~/Software/bcftools-1.3/bcftools view -H temp_tHaplSubset.vcf | awk '{print $2}'
> $(echo ${t}.PSEUDO.coordinates)
~/Software/bcftools-1.3/bcftools view -H temp_tHaplSubset.vcf |
awk '/synonymous_variant/||(/intergenic_region/&&!/protein_coding/) {print $2}'
> $(echo ${t}.PSEUDO.neutral.coordinates);

done
```

11 Controls for inferring topology from trees in non-recombined regions of the *t*-haplotype

We applied the pseudo-*t*-filter pipeline to the second half of Chr17. However, we allowed any SNP to be kept that was private to the *t*-carrier regardless of whether it was homozygous or heterozygous.

```
inFile=AllMouse_PASS_snps_maskedCNVs_Chr17-50-90Mb.vcf.gz

~/Software/bcftools-1.3/bcftools view -O z -s $(echo ${t},${WTs})
$inFile > tempSamps.vcf.gz

~/Software/bcftools-1.3/bcftools view -O z --private -s $(echo ${t})
tempSamps.vcf.gz > $(echo ${t}_Priv.vcf.gz)
```

As a second control, we extracted only private and heterozygous SNPs of *t*-carriers in the t complex:

```
inFile=AllMouse_PASS_snps_maskedCNVs_Tcomplex.vcf.gz

~/Software/bcftools-1.3/bcftools view -O z -s $(echo ${t},${WTs})
 $inFile > tempSamps.vcf.gz

~/Software/bcftools-1.3/bcftools view -O z --private -s $(echo ${t})
tempSamps.vcf.gz > tempPriv.vcf.gz
~/Software/bcftools-1.3/bcftools view -O z -e'GT="1/1" | GT="2/2" |
GT="3/3" | GT="."' tempPriv.vcf.gz > $(echo ${t}_PrivHET.vcf.gz)
```
