# Supplementary material for: Complex History and Differentiation Patterns of the t-Haplotype, a Mouse Meiotic Driver
Source: Genetics. 2017 Nov 14;208(1):365–75. doi: 10.1534/genetics.117.300513 (PMC5753869; doi:10.1534/genetics.117.300513)
Supplement: Supplementary file 2 [file 365FileS2.pdf]

Supplementary File 2

This document contains:

1. Figures S1-S10 (pages 2 to 15)
2. Table S1 (page 16)
3. A description of the Supplementary Data (pages 17 to 18)

Figure S1 (continues in next two pages): Figures 1-4 using coverage-filtered RAW SNPs (filtering procedure 2). A reanalysis using Harr et al.'s published raw SNP dataset, filtered based on coverage (see Materials and Methods). Panels A, B, C and D reproduce Figures 1, 2, 3 and 4, respectively.

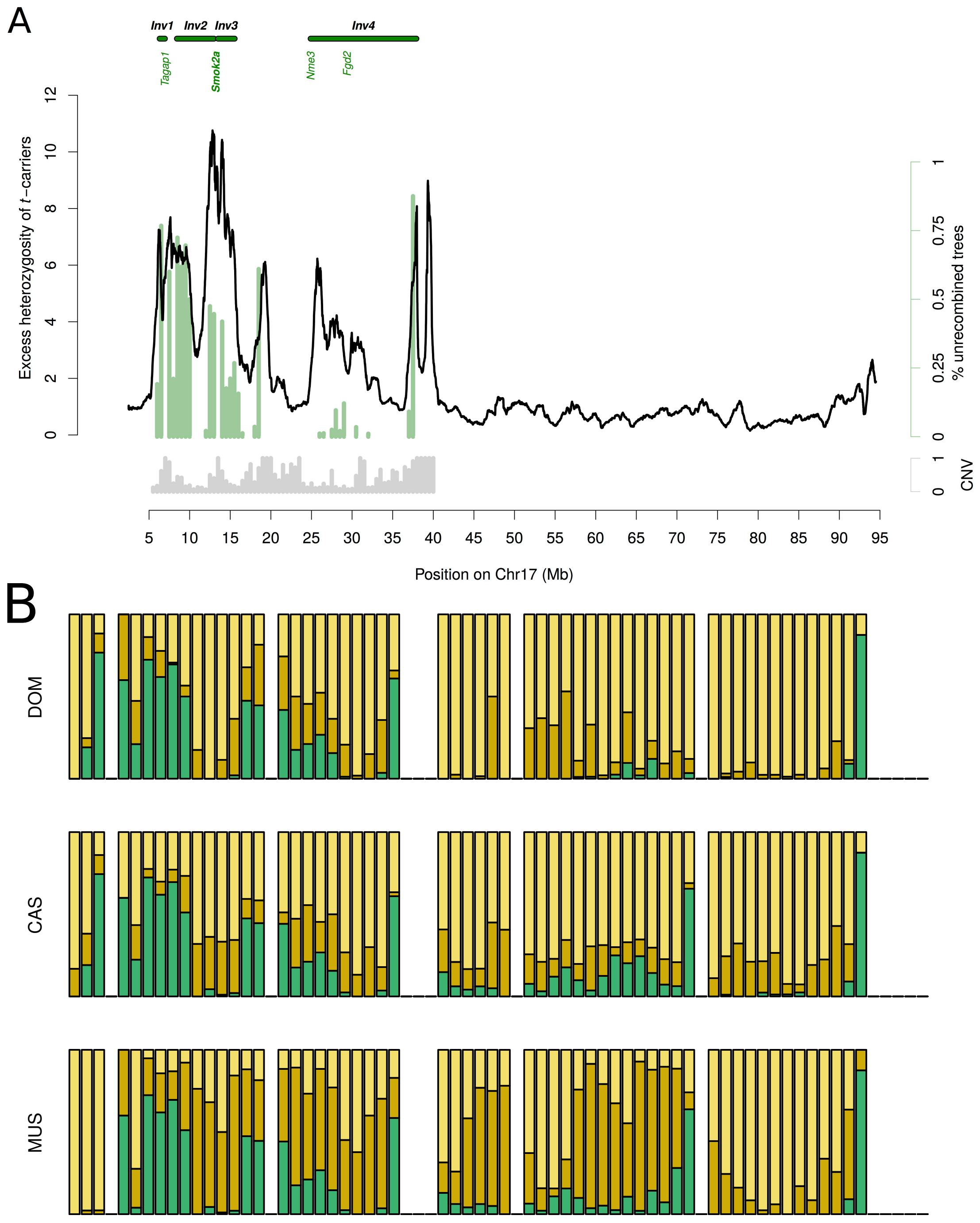

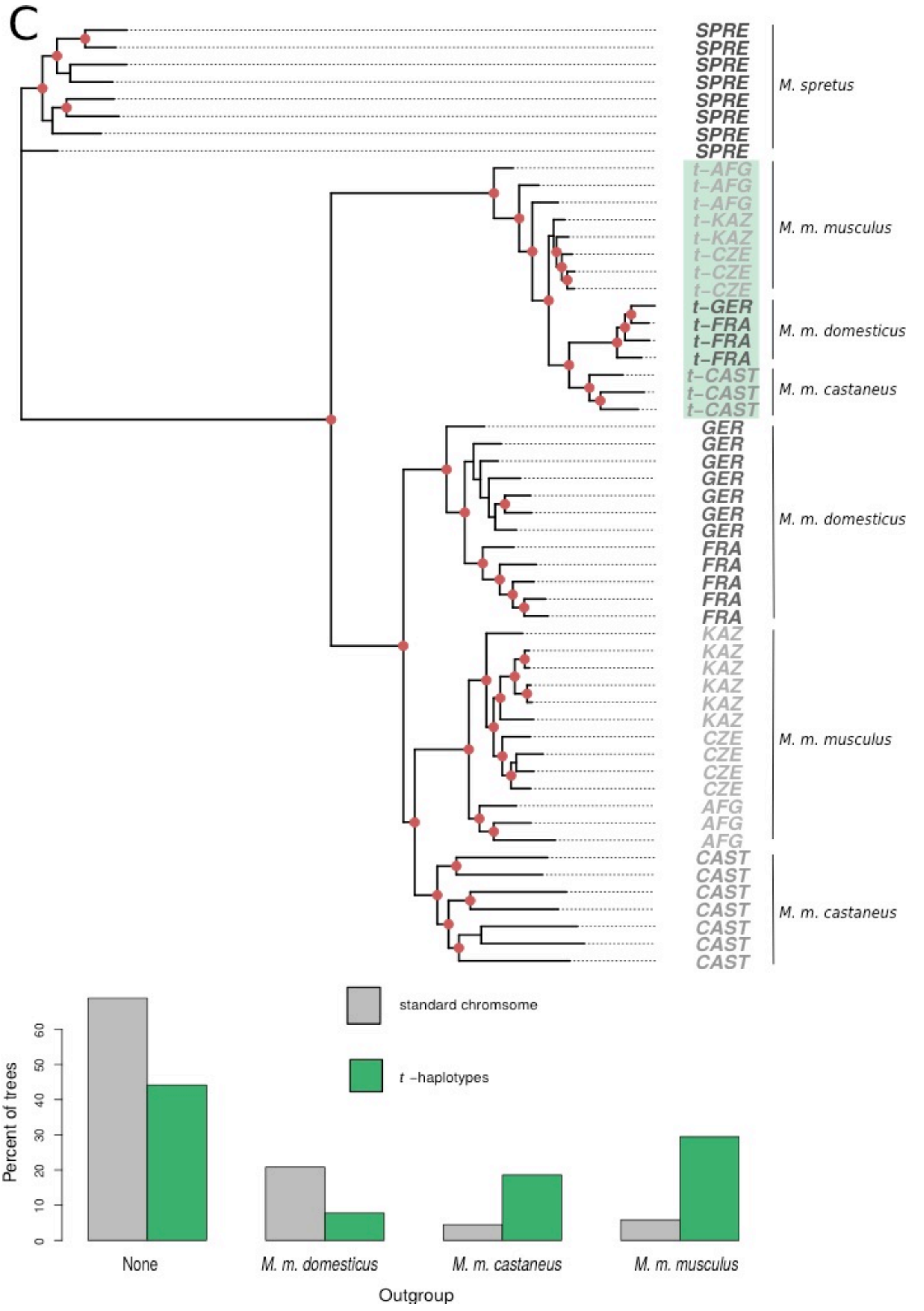
C
SPRE
M. spretus
t-AFG
t-KAZ
t-CZE
M. m. musculus
t-GER
t-FRA
M. m. domesticus
t-CAST
M. m. castaneus
GER
FRA
M. m. domesticus
KAZ
CZE
AFG
M. m. musculus
CAST
M. m. castaneus
standard chromsome
t -haplotypes
Percent of trees
0
10
20
30
40
50
60
None
M. m. domesticus
M. m. castaneus
M. m. musculus
Outgroup

D

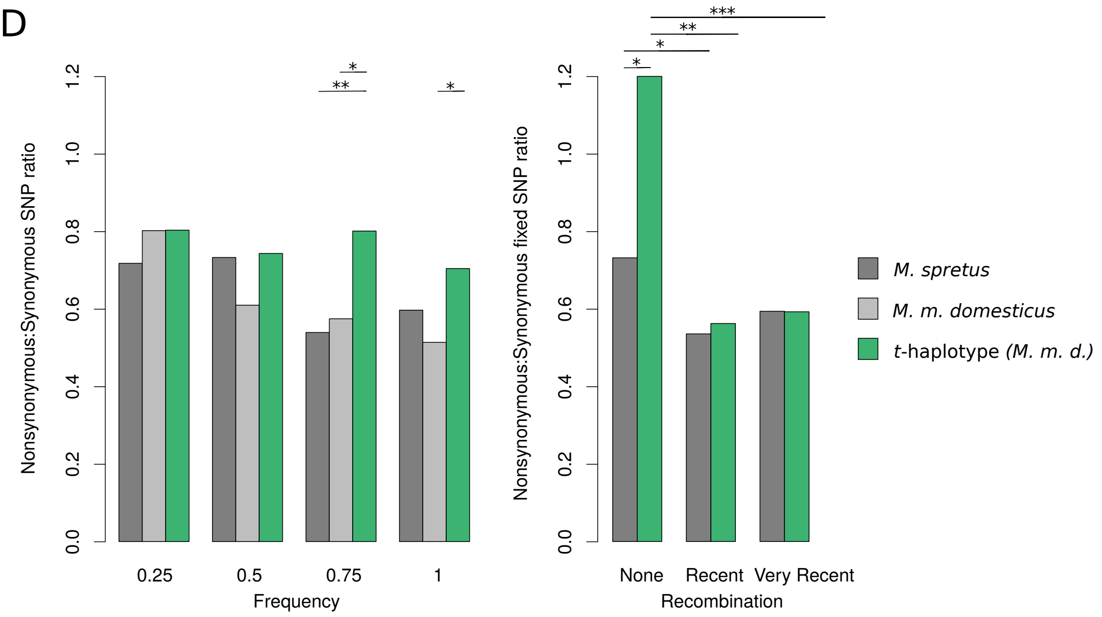

Nonsynonymous:Synonymous SNP ratio
0.0
0.2
0.4
0.6
0.8
1.0
1.2
**
*
*
0.25
0.5
0.75
1
Frequency
Nonsynonymous:Synonymous fixed SNP ratio
***
**
*
*
None
Recent
Very Recent
Recombination
M. spretus
M. m. domesticus
t-haplotype (M. m. d.)

Figure S2 (continues in the next two pages): Figures 1-4 using coverage- and allele-frequency-filtered RAW SNPs (filtering procedure 3). A reanalysis using Harr et al.'s published raw SNP dataset, filtered based on coverage and allele-frequency (see Materials and Methods). Panels A, B, C and D reproduce Figures 1, 2, 3 and 4, respectively.

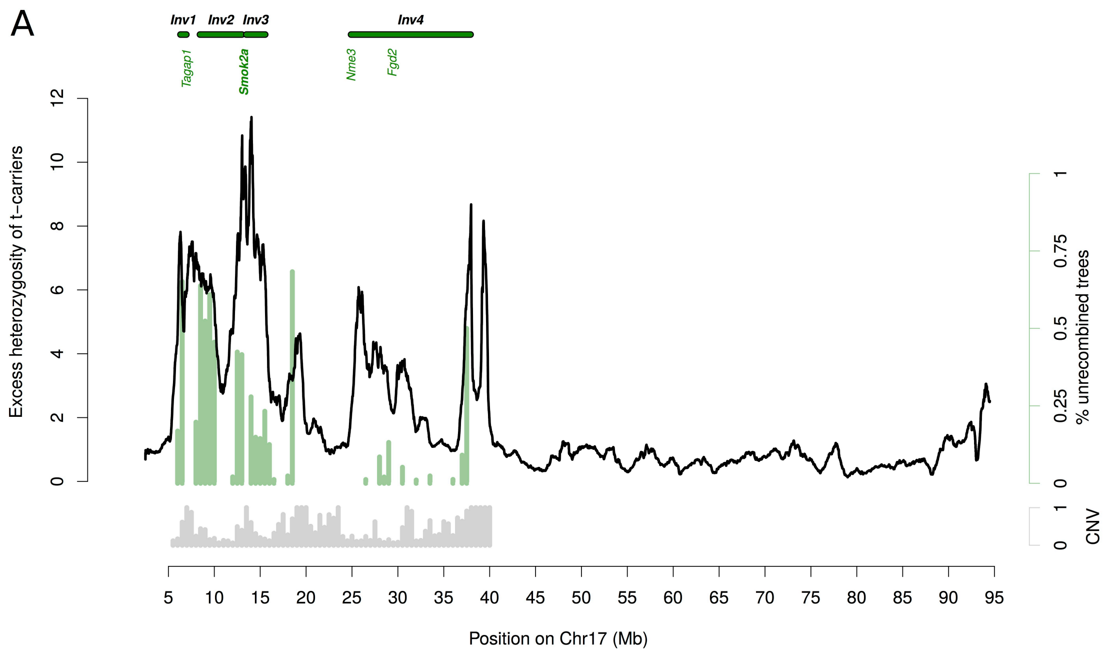

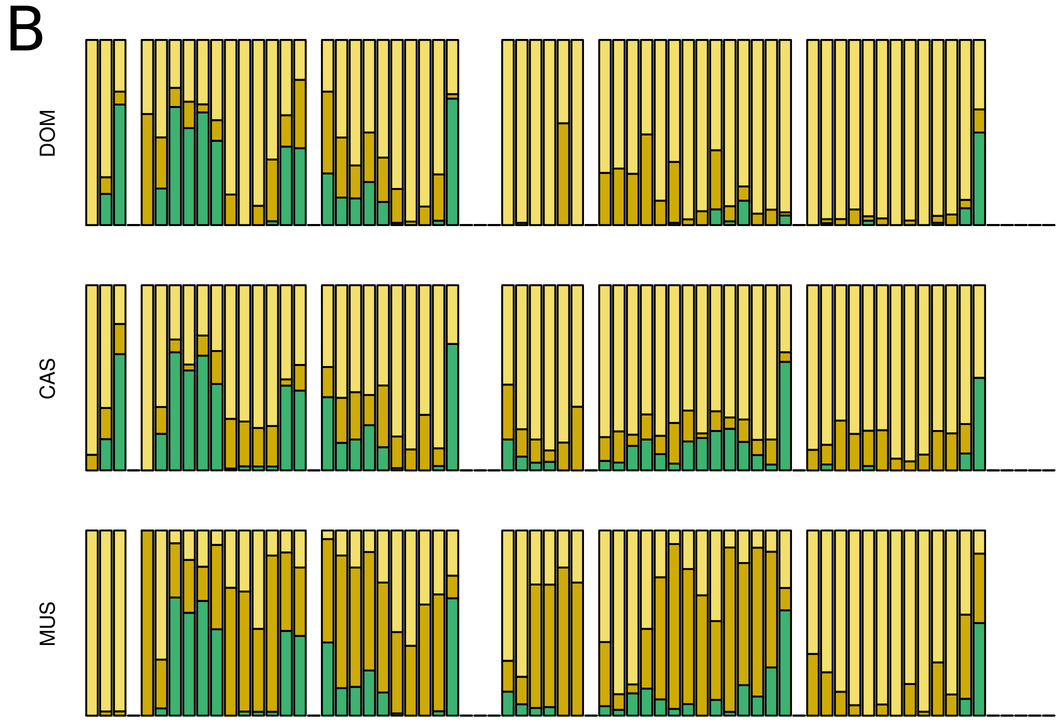

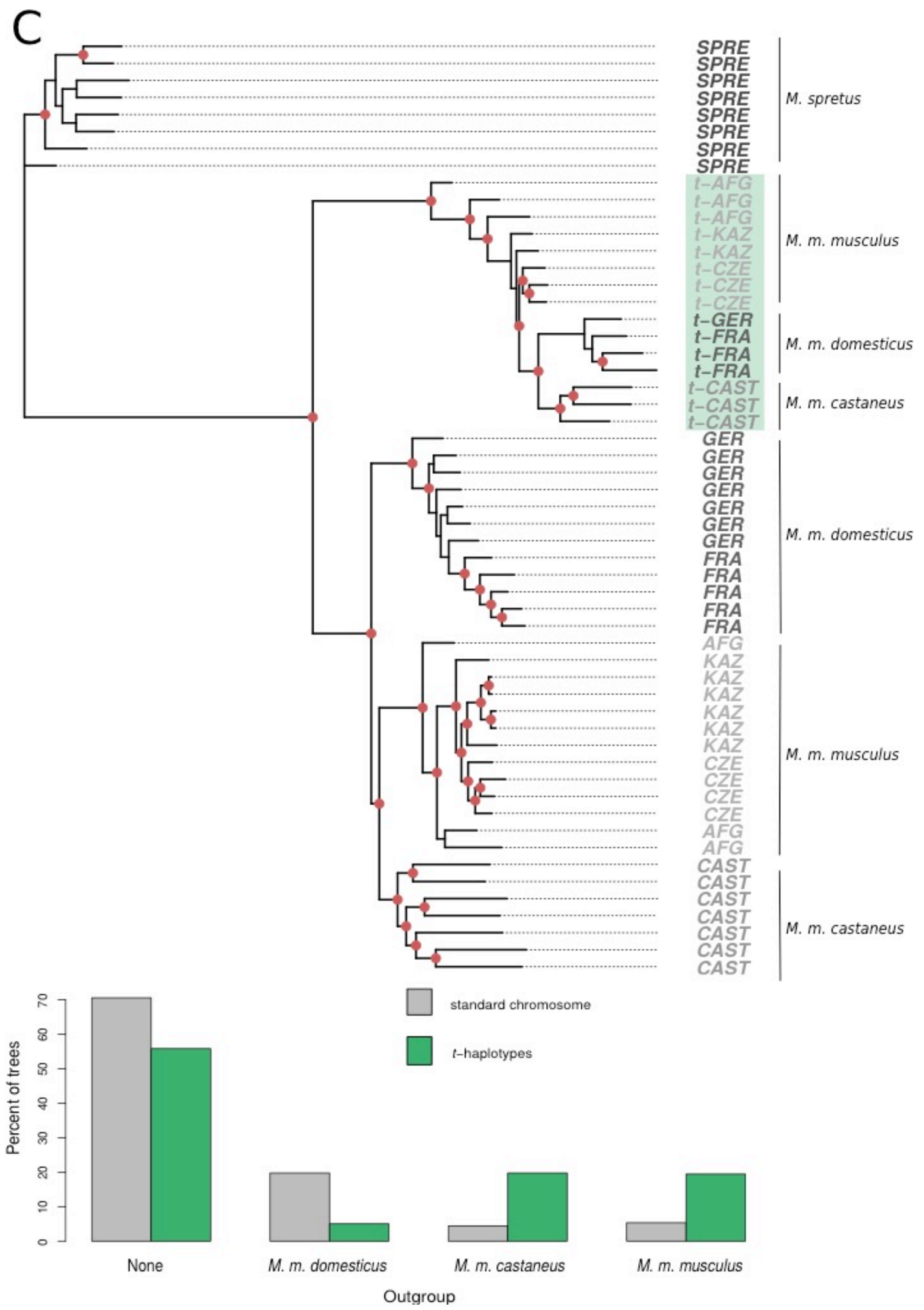
C
SPRE
SPRE
SPRE
SPRE
SPRE
SPRE
SPRE
SPRE
M. spretus
t-AFG
t-AFG
t-AFG
t-KAZ
t-KAZ
t-CZE
t-CZE
t-CZE
M. m. musculus
t-GER
t-FRA
t-FRA
t-FRA
M. m. domesticus
t-CAST
t-CAST
t-CAST
M. m. castaneus
GER
GER
GER
GER
GER
GER
GER
FRA
FRA
FRA
FRA
FRA
M. m. domesticus
AFG
KAZ
KAZ
KAZ
KAZ
KAZ
KAZ
CZE
CZE
CZE
CZE
AFG
AFG
M. m. musculus
CAST
CAST
CAST
CAST
CAST
CAST
CAST
M. m. castaneus
standard chromosome
t-haplotypes
Percent of trees
0
10
20
30
40
50
60
70
None
M. m. domesticus
M. m. castaneus
M. m. musculus
Outgroup

D

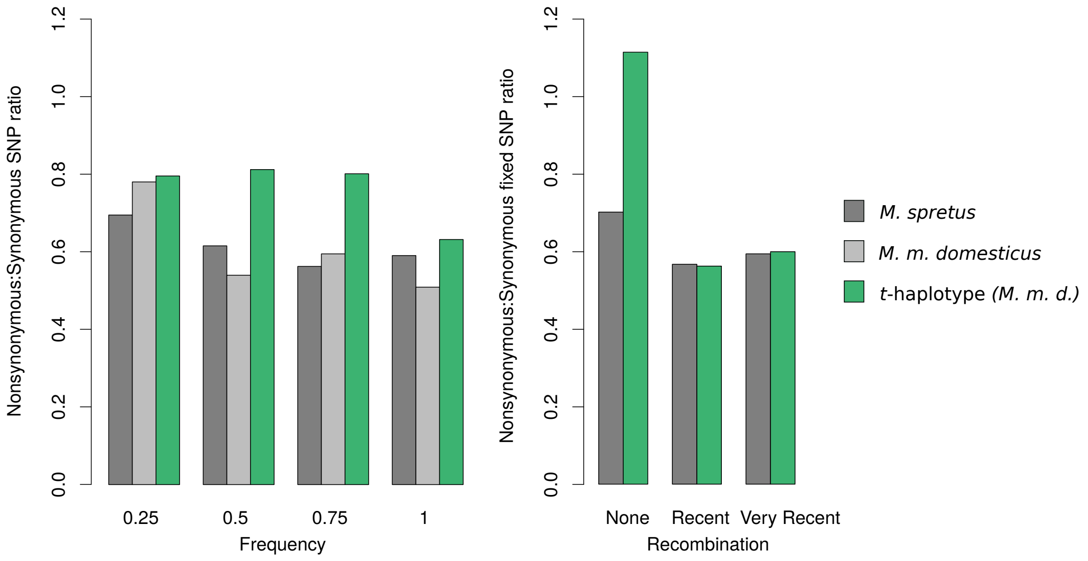

Nonsynonymous:Synonymous SNP ratio
0.0
0.2
0.4
0.6
0.8
1.0
1.2
0.25
0.5
0.75
1
Frequency
Nonsynonymous:Synonymous fixed SNP ratio
None
Recent
Very Recent
Recombination
M. spretus
M. m. domesticus
t-haplotype (M. m. d.)

Figure S3: SNP density in non-carrier *M. m. domesticus* individuals along chromosome 17. The average number of heterozygous SNPs per kilobase per individual is shown in windows of 1 Mb, sliding in steps of 1 Kb. All 12 non-carrier *M. m. domesticus* were used. Blue, purple and gray lines on the bottom of the plot mark genomic regions with large clusters of olfactory, immune and pheromone genes.

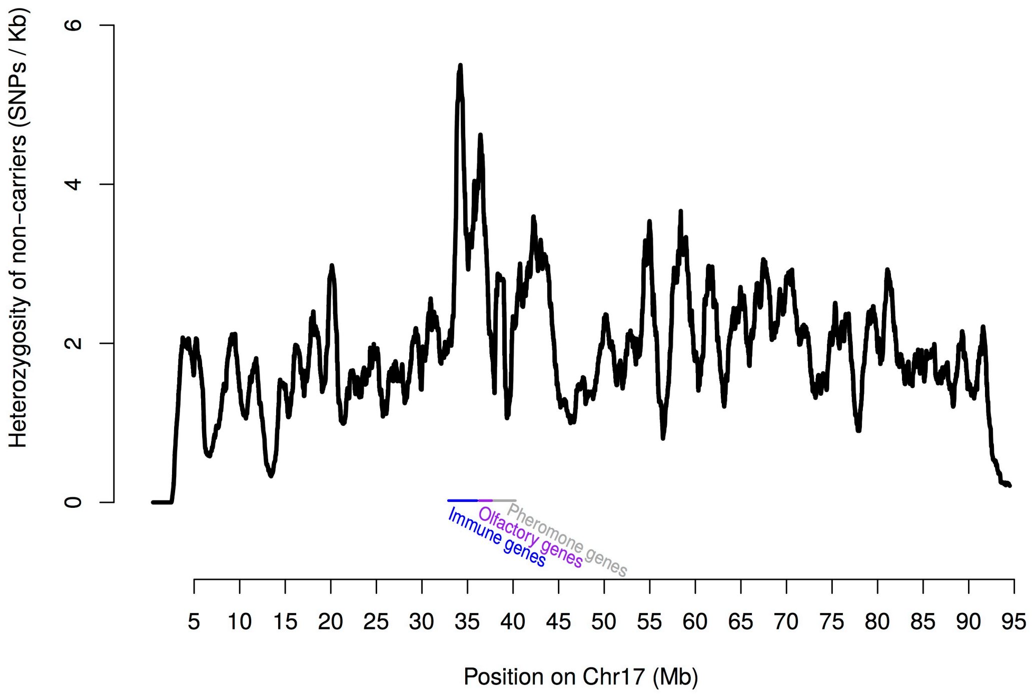

Figure S4: Divergence of the *t*-haplotype normalized by *M. m. domesticus* or *M. spretus* and using all, or only neutral SNPs. (A) Heterozygosity levels along the t-haplotype of *M. m. domesticus* estimated from all SNPs, and normalized by non-carrier heterozygosity (same as Figure 1). (B) Neutral heterozygosity levels along the t-haplotype of *M. m. domesticus*. The same procedure was followed as for Figure 1, but we only included intergenic or synonymous SNPs. (C) SNP density (per Kb) of pseudo-t-haplotypes normalized by that of M. spretus individuals. (D) Same as (C), but using only synonymous and intergenic SNPs. (E) SNP density (per Kb) of pseudo-t-haplotypes, *M. m. domesticus* non-carriers and *M. spretus* individuals. (F) Same as (E) but using only synonymous and intergenic SNPs. In all of the panels we used data for the entire chromosome 17 without masking CNVs.

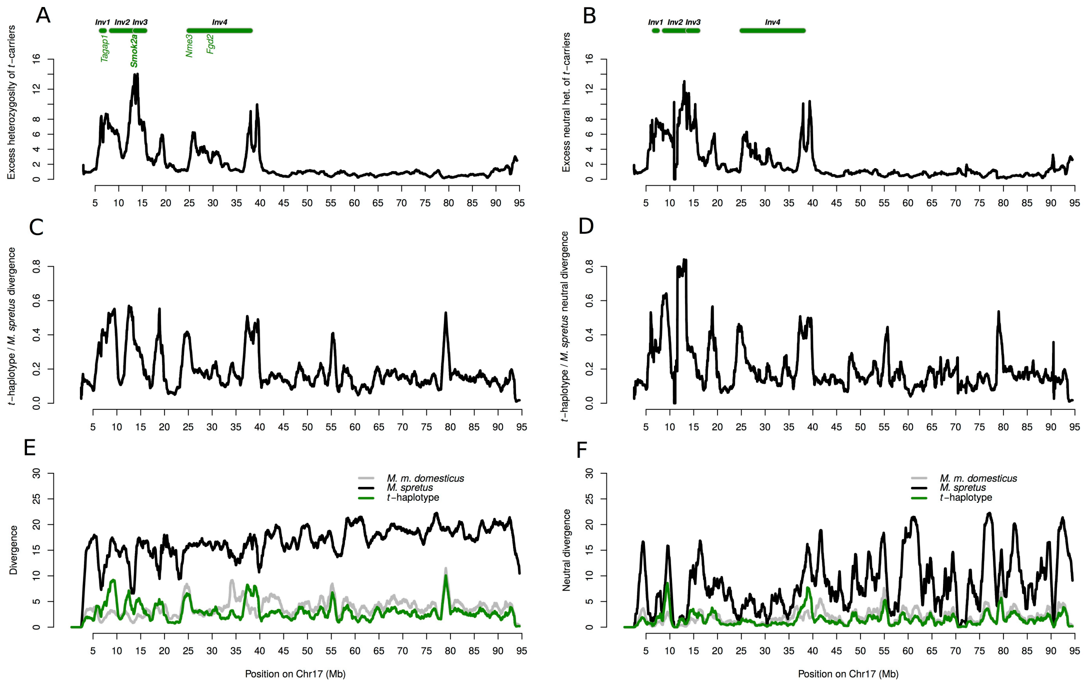

Figure S5: Divergence of the *t*-haplotype measured in abundance of insertions and deletions (INDELs). We plotted the ratio of INDEL density on chromosome 17 found in *t*-carriers compared to that found in non-carriers in *M. m. domesticus*. The densities were computed in windows of 1Kb, and the ratios were averaged over 1 Mb (sliding by 1Kb). We used the INDELs published in the raw SNP dataset of Harr et al. (2016). We plotted data for the entire chromosome 17 without masking CNVs.

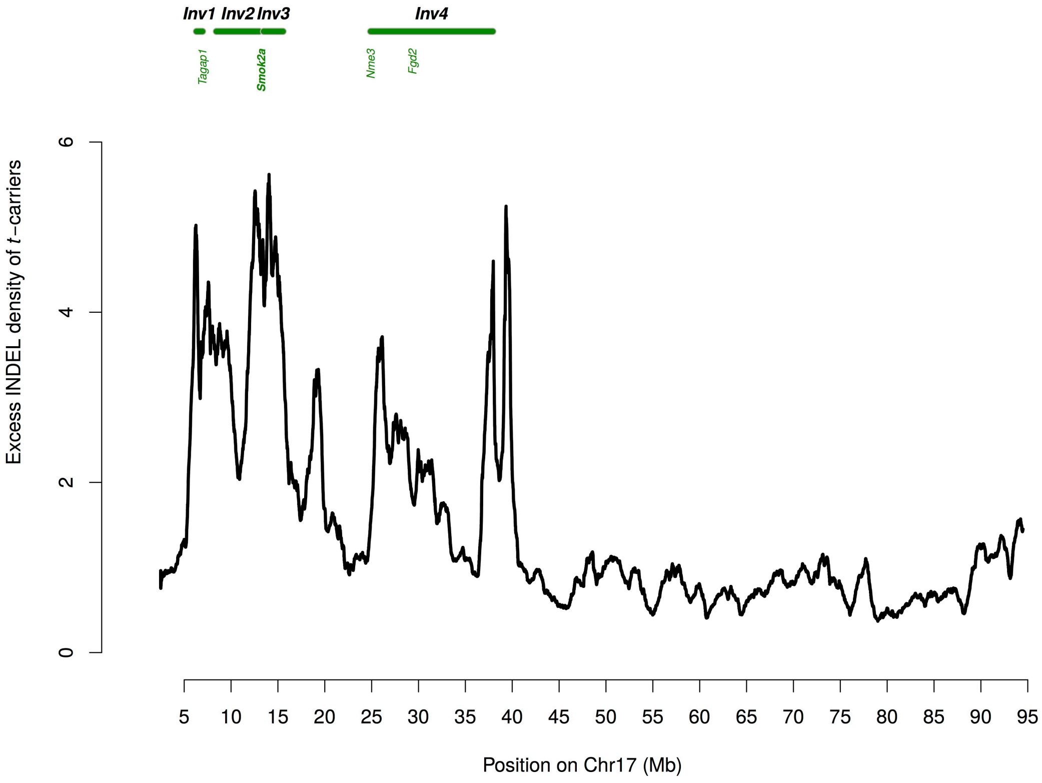

Figure S6: Alignment of the *tcp-1* region (exons 8-10) for standard chromosomes and *t*-haplotypes of *M. m. musculus*, *M. m. domesticus*, *M. m. castaneus* and *M. spretus*. Only sites that contain SNPs are shown. Sequences surrounded by red rectangles were generated by Morita et al. (1992) and retrieved from the NCBI nucleotide database (accessions X61222.1, X61212.1, X61219.1, X61217.1, X61215.1 and X61214.1). Other sequences were obtained from the variants provided in Harr et al. (2016). Arrows represent sites that were found in the original *t*-haplotype sequences but are not present on any of the standard *M. musculus* chromosomes. Black arrows represent the subset of these SNPs that are also detected on at least one pseudo-*t*-haplotype, red arrows mark SNPs that are not found in the pseudo-*t*-haplotypes.

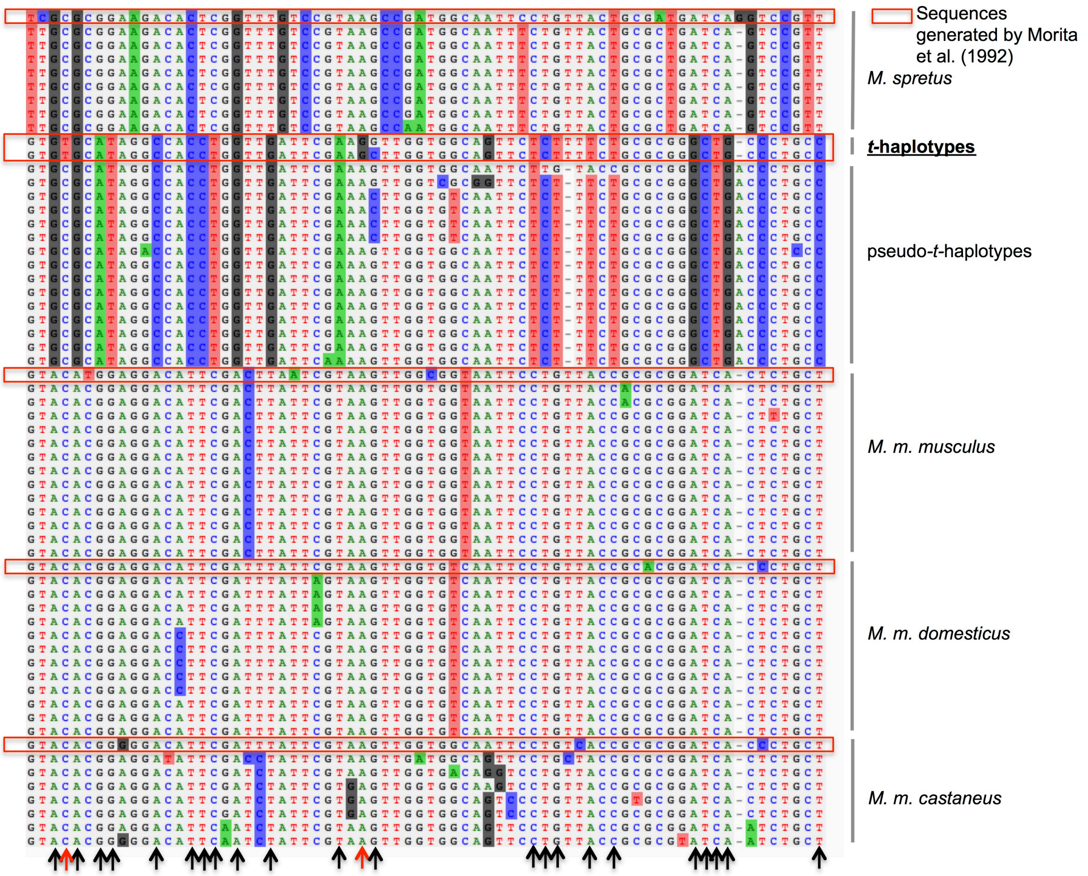

Figure S7: Phylogenetic topology along the t-haplotypes of the three subspecies using maximum parsimony (A) and neighbor joining (B) algorithm. The colors in the three bars represent the phylogenetic position of the "pseudo"-t-haplotypes from each of the three subspecies. The upper bar shows the results for *M. m. domesticus*, the middle bar for *M. m. musculus* and the lower bar for *M. m. castaneus*. Each segment in the bars corresponds to a 0.5 Mb window from 5-40 Mb on chromosome 17. The proportion of yellow, orange and green represent the proportion of the 5 Kb trees in the 0.5 Mb windows that show a certain topology. Yellow indicates that at least one t-haplotype is within its own subspecies, orange means that t-haplotypes are clustered within the *M. musculus* species complex but not within their respective subspecies, and green shows windows for which all t-haplotypes cluster outside of the *M. musculus* clade. DOM stands for *M. m. domesticus*, CAS for *M. m. castaneus* and MUS for *M. m. musculus*.

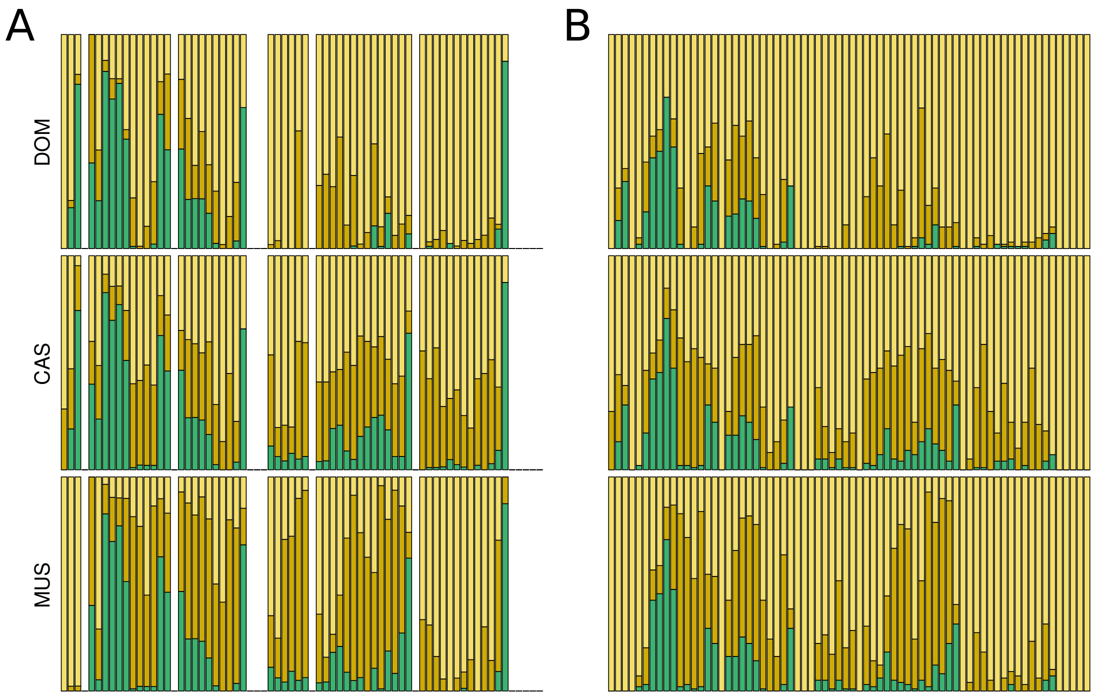

Figure S8: The topology of the *t*-haplotype phylogeny is robust to the removal of duplicated regions and to reconstruction based only on SNPs private to *t*-carriers. (A) Tree based on Chr. 17 region 50-90 Mb, which is outside of the *t* complex, using only SNPs that are private to *t*-carriers. (B) Tree based on all diverged regions (dark green bars in Figure 2 and Figure S7) for pseudo-*t*-haplotypes created using only SNPs that are private to *t*-carriers (independent of their homo/heterozygosity status). Red represents *M. m. castaneus*, orange *M. m. musculus*, yellow *M. m. domesticus*, and black *M. spretus*; sequences starting with "t-" refer to *t*-haplotypes. AFG, CZE and KAZ stand for *M. m. musculus* from Afghanistan, the Czech Republic and Kazakhstan, respectively, GER and FRA for *M. m. domesticus* from Germany and France, respectively, CAST stands for *M. m.* castaneus, and SPRE for *M. spretus*. (C) Percentage of 5 Kb regions in chromosome 17 region 50-90 Mb that support one subspecies being the outgroup to the other two. Grey bars represent the phylogeny of non-*t*-carriers, and green bars represent the phylogeny of *t*-carriers after retaining only private SNPs. (D) Percentage of 5 Kb regions in the non-recombined regions of the t complex that support one subspecies being the outgroup to the other two. Grey bars represent the phylogeny of non-*t*-carriers, and green bars represent the phylogeny of *t*-carriers after retaining only private SNPs.

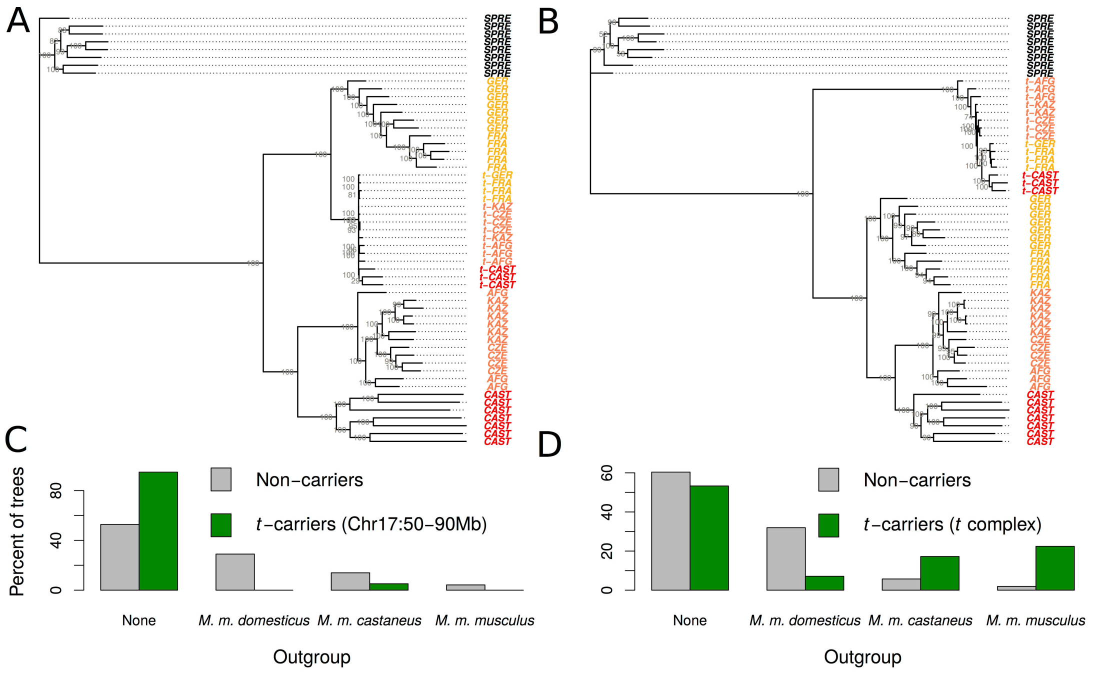

Figure S9: Percentage of trees supporting each possible arrangement of subspecies in the *t*-haplotype and non-carrier phylogeny using the maximum parsimony (A) and neighbor joining (B) algorithm. Percentage of trees based on 5 Kb windows of the region without recombination that show one subspecies as the outgroup to the others. Grey bars represent the phylogeny of non-*t*-carriers, and green bars represent the phylogeny of pseudo-*t*-haplotypes.

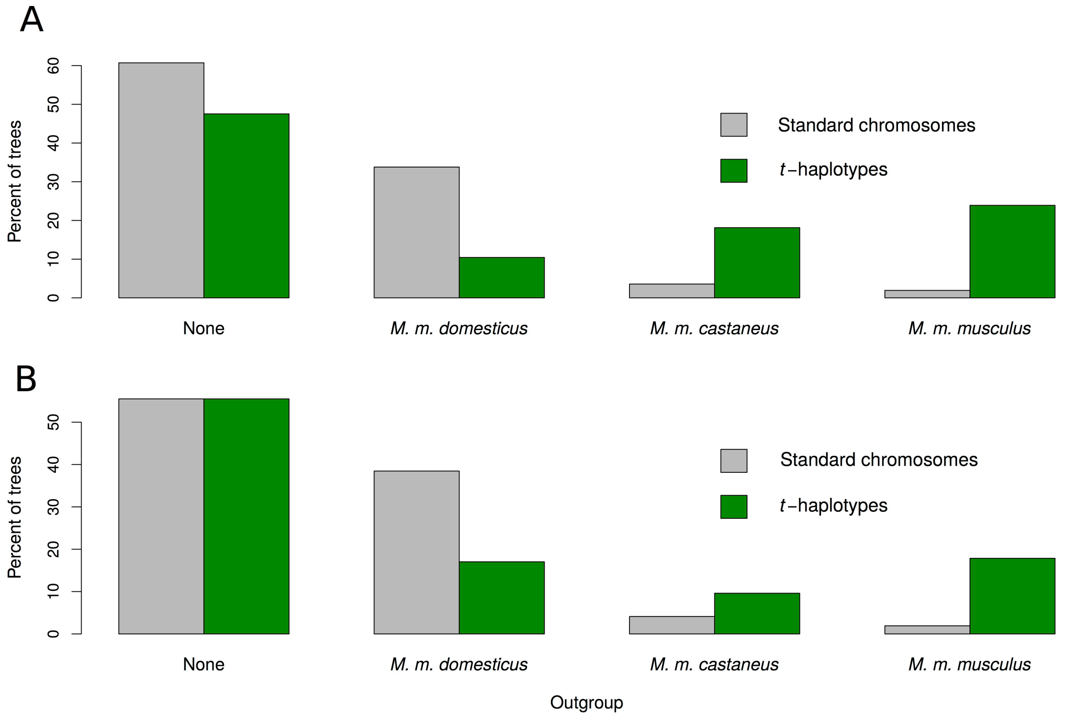

Figure S10: Divergence of gene expression between *t*-carriers and non carriers. (A-E) Percentage difference between the average gene expression of *t*-carrier and non-carriers (estimated as: | average_t-carrier - average_non-carrier | / average_non-carrier), plotted using a sliding window of 20 genes (using all genes with TPM>10 in non-carriers). Expression divergence is shown for (A) heart, (B) kidney, (C) muscle, (D) spleen, (E) thyroid. Regions that contain *t*-specific copy number variants (obtained by comparing the coverage of *t*-carriers to non-carriers, see Materials and Methods) are marked by grey rectangles. (F-J) Boxplots showing the percentage difference in expression of *t*-carriers relative to that of non-carriers for genes which overlap with at least 80% 5Kb windows for which no recombination was detected (green), some/old recombination was detected (orange) and recent/extensive recombination was detected (yellow), in (F) heart, (G) kidney, (H) muscle, (I) spleen, (J) thyroid. (K-O) Boxplots showing the percentage difference in expression of *t*-carriers relative to that of non-carriers for genes overlapping or not overlapping a CNV, in (K) heart, (L) kidney, (M) muscle, (N) spleen, (O) thyroid.

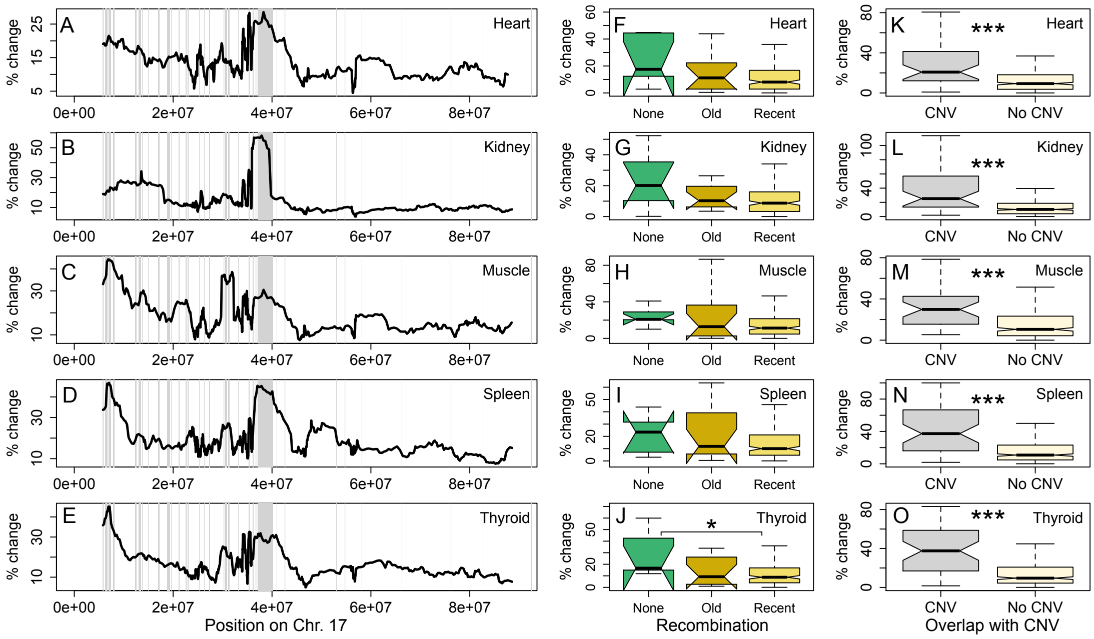

Table S1: Differentially expressed transcripts between *M. m. domesticus* wild type individuals and *t*-carriers in testis, heart, kidney, brain, thyroid, muscle, spleen and liver.

**Brain**					
**Gene_ID**	**Gene Name**	**Chrom.**	**Non-carrier Average TPM**	**t-carrier Average TPM**	**q-value**
ENSMUSG00000000579	Dynlt1c	17	36.6	81.7	0.04122
ENSMUSG00000026269	Rnpepl1	1	345.6	386.1	0.00861
ENSMUSG00000036315	Znrd1	17	94.1	158.9	0.04122
ENSMUSG00000056692	D17Wsu92e	17	749.7	991.1	0.04122
ENSMUSG00000071984	Fndc1	17	240.2	76.3	0.00861
ENSMUSG00000092074	Dynlt1a	17	36.6	81.7	0.04122
ENSMUSG00000095677	Dynlt1f	17	36.6	81.7	0.04122

**Heart**					
No significantly expressed genes found.					

**Kidney**					
**Gene_ID**	**Gene Name**	**Chrom.**	**Non-carrier Average TPM**	**t-carrier Average TPM**	**q-value**
ENSMUSG00000023828	Slc22a3	17	9.9	71.0	0.00129
ENSMUSG00000024032	Tff1	17	56.6	23.4	0.03007

**Liver**					
**Gene_ID**	**Gene Name**	**Chrom.**	**Non-carrier Average TPM**	**t-carrier Average TPM**	**q-value**
ENSMUSG00000079707	Tcte3	17	6.7	40.1	0.04567

**Spleen**					
**Gene_ID**	**Gene Name**	**Chrom.**	**Non-carrier Average TPM**	**t-carrier Average TPM**	**q-value**
ENSMUSG00000033450	Tagap	17	305.6	793.1	0.01156

**Testis**					
**Gene_ID**	**Gene Name**	**Chrom.**	**Non-carrier Average TPM**	**t-carrier Average TPM**	**q-value**
ENSMUSG00000000579	Dynlt1c	17	1230.5	5557.4	6.76E-08
ENSMUSG00000014956	Ppp1cb	5	281.3	2679.9	6.61E-08
ENSMUSG00000023828	Slc22a3	17	8.2	108.1	3.86E-06
ENSMUSG00000029265	Dr1	5	531.2	802.1	0.00115
ENSMUSG00000036214	Znrd1as	17	782.0	3106.3	6.61E-08
ENSMUSG00000040188	Scamp2	9	1285.4	920.4	0.00140
ENSMUSG00000046711	Hmga1	17	384.4	587.7	0.00102
ENSMUSG00000055602	Tcp10b	17	1259.7	2275.7	0.01609
ENSMUSG00000059030	Olfr128	17	51.3	24.4	0.03348
ENSMUSG00000068037	Mas1	17	344.9	157.9	0.00171
ENSMUSG00000092074	Dynlt1a	17	1230.5	5557.4	6.76E-08
ENSMUSG00000095677	Dynlt1f	17	1230.5	5557.4	6.76E-08

**Thyroid**					
**Gene_ID**	**Gene Name**	**Chrom.**	**Non-carrier Average TPM**	**t-carrier Average TPM**	**q-value**
ENSMUSG00000026269	Rnpepl1	1	464.9	578.5	0.00020

**Muscle**					
**Gene_ID**	**Gene Name**	**Chrom.**	**Non-carrier Average TPM**	**t-carrier Average TPM**	**q-value**
ENSMUSG00000073471	Rsph3a	17	310.2	717.2	0.00886

Description of the Supplementary Data

All the Supplementary Data are available at:
http://dx.doi.org/10.15479/AT:ISTA:78

##1. Content of folders:
"1-PASS_SNPs for the first filtering"
"2-Coverage-and_AlleleRatio-Filtered_RAW_SNPs"
"3-Coverage-Filtered_RAW_SNPs"

For each of these three SNP filtering procedures, we provide:

1-Trees_for_all_5kb_windows : the trees obtained for each 5Kb window using Maximum Likelyhood (ML_trees.zip), Neighbor-Joining (NJ_trees.zip), and Maximum Parsimony (MP_trees.zip).

2-Tree_topologies_for_all_5kb_windows : The topologies obtained for each tree and species. The topology files (e.g. Tree_results_cas_sorted) contain the following collumns:

1. Location on the t-complex. This corresponds to the location on Chromosome 17 minus 5000000 (for instance, the first window, 0-5000, is located on chromosome 17 from 5000000 to 5050000).
2. The status of t-haplotypes relative to their subspecies: 1 = nested, 0 = outgroup.
3. The status of t-haplotypes relative to the M. musculus species complex: 1 = nested, 0 = outgroup.
4. The topological color of the tree: 0 = no recombination (dark green), 1 = old recombination (light green), 2 = recent recombination (orange).

3-TreeFile_Concatenated_Non-Recombined_Regions : the tree obtained (with IQ-Tree, Maximum Likelihood) from the concatenated non-recombining regions.

4-Divergence_SNPcoordinates : The coordinates of all SNPs used to produce Figure 1 and Figure S4. The folder "Main" contains the coordinates of only heterozygous SNPs (Figure 1). The lists of heterozygous SNP locations for each *M. m. domesticus* individual on chromosome 17 are contained in a separate file named according to the respective mouse ID (see Supplementary Methods in File S1). The folder named "Controls" contains further folders corresponding to the neutral heterozygosity figure (heterozygous SNPs that are either synonymous or intergenic; used for Figure S4B), the pseudo-t vs. *M. spretus* figure (all SNPs found in pseudo-t-haplotypes and in *M. spretus* individuals; used for Figure S4C and E), and to the neutral pseudo-*t* vs *M. spretus* figure (heterozygous SNPs found in pseudo-*t*-haplotypes and *M. spretus* individuals, which are either synonymous or intergenic; used for Figure S4D and E). Each file contains a list of SNP coordinates for chromosome 17 for the individual indicated in the name of the file.

5-Deterioration_SNPcoordinates : The coordinates of all homozygous and heterozygous SNPs that are either missense or synonymous (used to plot Figure

4). The folder DOM_NonT contains SNP locations for non-carrier individuals from *M. m. domesticus*, the folder DOM_T contains SNP locations for pseudo-*t*-haplotypes *M. m. domesticus* individuals, while the folder SPRET contains SNP locations for *M. spretus* individuals. Each file is named in the following way: individual_ID.heterozygous/homozygous.missense/synonymous and contains the list of coordinates in the region chr17:5-40 Mb.

##2. Content of folder "4-Expression"
All the final gene expression values, as well as the corresponding q-values, are provided in the folder 4-Expression (one file per tissue).

##3. Content of folder "5-CNV-regions"
A list of all the identified CNVs is provided in the folder 5-CNV-regions.
